# Chemical Constituents of the Flowers of *Pueraria lobata* and Their Cytotoxic Properties

**DOI:** 10.3390/plants11131651

**Published:** 2022-06-22

**Authors:** Yejin Kim, Jaeyoon Kim, So-Ri Son, Ji-Young Kim, Jung-Hye Choi, Dae Sik Jang

**Affiliations:** Department of Biomedical and Pharmaceutical Sciences, Graduate School, Kyung Hee University, Seoul 02447, Korea; yezeen@khu.ac.kr (Y.K.); yoonie1028@khu.ac.kr (J.K.); allosori@khu.ac.kr (S.-R.S.); k_christina@khu.ac.kr (J.-Y.K.); jchoi@khu.ac.kr (J.-H.C.)

**Keywords:** *Pueraria lobata*, Puerariae Flos, tryptophan derivatives, flavonoids, cytotoxicity, ovarian cancer

## Abstract

The flower of *Pueraria lobata* (Puerariae Flos) is a reddish-purple to violet-purple flower that blooms between July and September. In our preliminary study, Puerariae Flos extract exhibited significant activity against a human ovarian cancer cell line. This research aims to identify the active compounds in Pueraria Flos. By repeated chromatography, one new tryptophan derivative (**1**), two new flavanones (**4** and **5**), and 19 known compounds, including tryptophan derivatives (**2** and **3**), flavonoids (**6**–**9**), isoflavonoids (**10**–**20**), a flavonolignan (**21**), and a phenolic compound (**22**), were isolated from a methanol extract of Puerariae Flos. The structures of new compounds were elucidated as 13-*N*-benzoyl-l-tryptophan-1-*N-β*-d-glucopyranoside (**1**), 2-hydroxy-5-methoxy-naringenin (**4**), and 2-hydroxy-5-methoxy-naringenin 7-*O*-*β*-d-glucopyranoside (**5**). Among the isolates, afromosin (**17**), tectorigenin (**11**), apigenin (**8**), glycitein (**16**), (-)-hydnocarpin (**21**), irilin D (**12**), irisolidone 7-*O*-glucoside (**14**), and genistein (**10**) showed cytotoxicity against human ovarian cancer cell line A2780. Apigenin (**8**) and (-)-hydnocarpin (**21**) were the most active (IC_50_ values of 9.99 and 7.36 μM, respectively).

## 1. Introduction

*Pueraria lobata* (Willd.) Ohwi (Leguminosae) is one of the most important Chinese traditional medicines native to East Asia, Central and South America, and Europe [1]. The flower of *P. lobata* (Puerariae Flos) is a reddish-purple to violet-purple flower that blooms between late July and September [2]. It has been commonly used as a hangover treatment in traditional oriental medicine due to its enhancing activity of acetaldehyde removal [3,4]. Puerariae Flos mainly contains isoflavonoids, flavonoids, saponins, tryptophan derivatives, and phenolic compounds [5,6,7,8]. Extracts and secondary metabolites from Puerariae Flos have shown potential pharmacological effects, such as an anti-diabetic effect [9,10], an anti-inflammatory effect [11], estrogen-like activity [12], an anti-cancer property [12], an anti-endometriotic effect [13], and a sensitizing effect on paclitaxel-resistant ovarian cancer cells [14].

Ovarian epithelial cancer is one of the most fatal cancers in women [15]. A previous investigation has shown that the 5-year relative survival rate for patients with ovarian epithelial cancer was only 30% [16]. Platinum–taxane chemotherapy after surgical resection is the most usual treatment for ovarian cancer patients. However, tumor cell heterogeneity causes treatment resistance through multiple genetic alterations, leading many patients to relapse or even die [17]. As a result, a novel therapeutic agent for ovarian cancer is urgently needed. Numerous plant-derived medicines and their derivatives induce apoptosis in ovarian carcinoma cell lines [18]. The genus *Pueraria* has shown cytotoxic and anti-proliferative activity against various cancer cells [11]. In particular, the phytoestrogens extracted from Puerariae rhizome exhibited anti-proliferative activity against ovarian cancer cells [14]. In our preliminary experiment, a methanol extract of Puerariae Flos exhibited a significant cytotoxic effect against the human ovarian cancer cell line A2780. Therefore, the purpose of this study is to find active compounds in Pueraria Flos against human ovarian cancer cells.

Herein, repeated chromatography with the methanol extract of Puerariae Flos was conducted to isolate compounds with cytotoxic activity against human ovarian cancer cell line A2780. The structure of isolated compounds was determined by analyzing 1D- and 2D-nuclear magnetic resonance (NMR) spectroscopic data and high-resolution mass spectroscopy (HRMS). Then, the 3-(4,5-dimethylthiazol-2-yl)-2,5-diphenyl tetrazolium bromide (MTT) assay was used to assess the cytotoxicity of all isolated compounds (**1**−**22**) against human epithelial ovarian cancer cell line A2780 and the immortalized ovarian epithelial cell line IOSE80PC.

## 2. Results and Discussion

### 2.1. Sturcture Elucidation of Isolated Compounds

**Figure 1:**
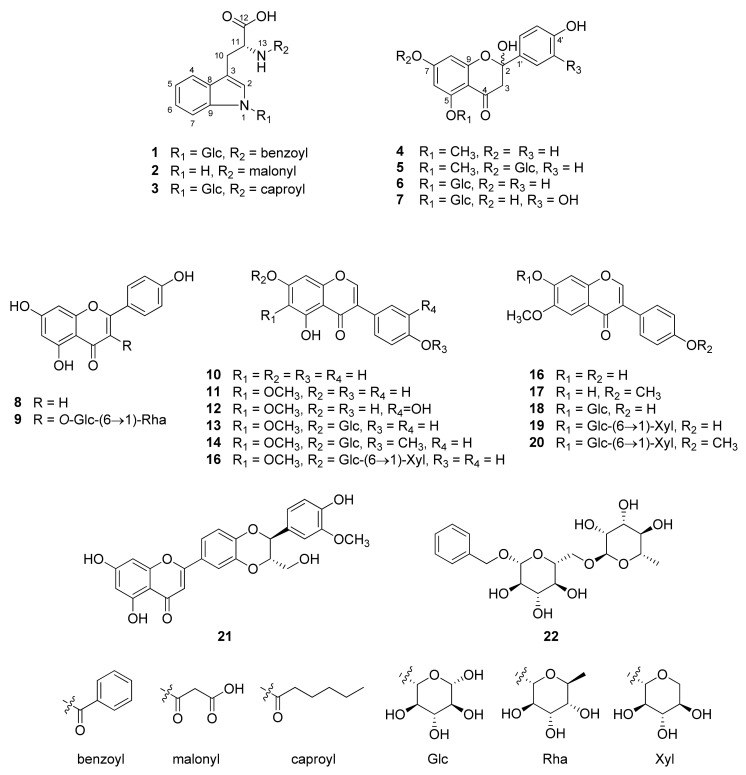
Structures of compounds **1**–**22** isolated from Puerariae Flos.

In the present study, one new tryptophan derivative (**1**), two new flavanones (**4** and **5**), and 19 known compounds (**2**–**3** and **6**–**22**) were isolated from the methanol extract of Pueariae Flos (Figure 1).

**Figure 2:**
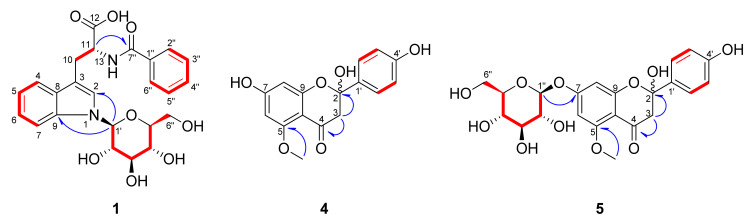
Key ^1^H-^1^H COSY (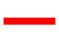) and ^1^H-^13^C HMBC (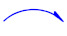) correlations for the new compounds **1**, **4**, and **5**.

Compound **1** was isolated as a pale-yellow powder. The molecular formula of **1** was established as C_24_H_26_N_2_O_8_ by its positive precursor ion in the high-resolution electrospray ionization orbitrap mass spectrum (HR-ESI-Orbitrap-MS; *m*/*z* = 471.1758 [M+H]^+^; calculated for C_24_H_27_N_2_O_8_, 471.1762) (Figure S1). The infrared (IR) absorption spectrum revealed the presence of conjugated carbonyl (1718 cm^−1^) and amide (1630 cm^−1^) groups (Figure S22). The ^1^H-NMR spectrum of **1** revealed five olefinic signals (*δ*_H_ 6.92 (1H, ddd, *J* = 8.0, 7.0, 1.0 Hz, H-5), 7.10 (1H, ddd, *J* = 8.0, 7.0, 1.0 Hz, H-6), 7.27 (1H, s, H-2) 7.47 (1H, d, *J* = 7.0 Hz, H-7), and 7.52 (1H, d, *J* = 8.0 Hz, H-4)), a methine signal (*δ*_H_ 4.78 (1H, t, *J* = 5.0 Hz, H-11)), and signals for a methylene moiety (*δ*_H_ 3.34 (1H, overlapped, H-10a) and 3.49 (1H, overlapped, H-10b)) (Table 1, Figure S2). In addition, by observing the chemical shift and the vicinal coupling constant of an anomeric proton signal (*δ*_H_ 5.41 (1H, d, *J* = 9.0 Hz, H-Glc-1′)) and the signals of *δ*_H_ 3.49—3.92 ppm, we expected that **1** has a *β*-glucose moiety. It also revealed signals for a benzoyl residue at *δ*_H_ 7.38 (2H, dd, *J* = 8.5, 7.5 Hz, H-3″, 5″), 7.46 (1H, tt, *J* = 7.5, 1.5 Hz, H-4″), and 7.67 (2H, dd, *J* = 8.5, 1.5 Hz, H-2″, 6″) in the ^1^H-NMR spectrum. Compared to previously reported data, the chemical structure of **1** was very similar to that of tryptophan-*N*-glucoside, except for the presence of a benzoyl aromatic ring [19]. Through the ^13^C-NMR spectrum of **1**, 24 carbon signals were observed including ten olefinic (*δ*_C_ 111.8, 120.0, 121.1, 123.2, 125.5, 128.6 x2, 129.7 x2, and 132.9), four quaternary (*δ*_C_ 112.7, 130.4, 135.6, and 138.5), two carboxylic (*δ*_C_ 170.4 and 175.9), two methylene (*δ*_C_ 28.3 and 62.9), and six methine carbon signals including an anomeric carbon (*δ*_C_ 55.6, 71.6, 73.8, 79.1, 80.7, and 86.8) (Table 1, Figure S3). By analysis of the coherence spectroscopy (COSY) NMR spectrum, the connections from H-4 to H-7, H-2” to H-6”, H-Glc-1′ to H-Glc-6′, and H-10 to H-11 were revealed (Figure 2 and Figure S5). The positions of the *β*-glucopyranosyl and benzoyl groups were determined to be N-1 and N-13, respectively, by analysis of the correlations in the ^1^H-^13^C heteronuclear multiple bond correlation (HMBC) NMR spectrum from H-Glc-1′ (*δ*_H_ 5.41) to C-3 (*δ*_C_ 112.7)/C-9 (*δ*_C_ 138.5) and from H-11 (*δ*_H_ 4.78) to C-7″ (*δ*_C_ 170.4) (Figure 2 and Figure S6). The absolute configuration of tryptophan moiety was confirmed as l-tryptophan by an acid hydrolysis and measurement of optical rotation. Furthermore, the sugar of **1** was established as *β*-d-glucopyranose by an acid hydrolysis and high-performance liquid chromatography (HPLC) analysis (Figure S25). According to these data, the new compound **1** was elucidated as 13-*N*-benzoyl-l-tryptophan-1-*N*-*β*-d-glucopyranoside.

Compound **4** was obtained as a brown powder. The molecular formula of **4** was established as C_16_H_14_O_6_ by its positive precursor ion in the high-resolution quadrupole time-of-flight mass spectrum (HR-Q-TOF-MS; *m*/*z* = 303.0879 [M+H]^+^; calculated for C_16_H_15_O_6_, 303.0864) (Figure S8). The IR spectrum showed the presence of the conjugated carbonyl (1676 cm^−1^) and benzene ring (1588, 1510 cm^−1^) (Figure S23). The ^1^H-NMR spectrum of **4** exhibited two resonances of AA’BB’ spin systems of an aromatic ring (*δ*_H_ 7.05 (2H, d, *J* = 8.5 Hz, H-3′, 5′) and 7.57 (2H, d, *J* = 8.5 Hz, H-2′, 6′)], two *meta* coupled proton signals (*δ*_H_ 6.12 (1H, d, *J* = 1.5 Hz, H-6) and 6.49 (1H, d, *J* = 1.5 Hz, )-8)), a methylene signal at *δ*_H_ 3.66 (2H, q, *J* = 14.0 Hz, H-3), and a methoxy signal at *δ*_H_ 3.56 (3H, s) (Table 2, Figure S9), indicating that **4** is a flavanone with a methoxy group (Figure S9a) [20]. The ^13^C- and DEPT NMR spectra showed 16 characteristic carbon signals for 2-hydroxyflavanone with a methoxy group; two oxygenated aromatic carbon signals at *δ*_C_ 102.1 and 173.5, two aromatic quaternary carbon signals at *δ*_C_ 160.6 and 174.2, two aromatic methine carbon signals at *δ*_C_ 92.7 and 94.8 of the A ring, four aromatic methine carbon signals at *δ*_C_ 116.2 × 2 and 132.9 × 2, an oxygenated aromatic carbon signal at *δ*_C_ 158.1, an aromatic quaternary carbon signal at *δ*_C_ 126.1 of the B ring, a carbonyl carbon signal at *δ*_C_ 194.3, a hemiketal carbon signal at *δ*_C_ 107.6, a methylene carbon signal at *δ*_C_ 42.4 of the C ring, and a methoxy carbon signal at *δ*_C_ 55.7 (Table 2, Figure S10). The location of the methoxy group was determined at C-5 by a correlation in the HMBC NMR spectrum from H-OCH_3_ (*δ*_H_ 3.56) to C-5 (*δ*_C_ 160.6) (Figure 2 and Figure S13). The relative stereochemistry of the hydroxy group at C-2 was not determined due to the inconsistent value of the optical rotation. Accordingly, it was expected that the *R* and *S* configuration would coexist. Attempts were made to separate the enantiomers but failed. Thus, the structure of **4** was elucidated as 2-hydroxy-5-methoxy-naringenin.

Compound **5** was obtained as a brown powder. The molecular formula of **5** was established as C_22_H_24_O_11_ from the HR-ESI-Orbitrap-MS (*m*/*z* = 465.1391 [M+H]^+^, calculated for C_22_H_25_O_11_, 465.1392) (Figure S15). The IR spectrum showed the presence of the conjugated carbonyl (1687 cm^−1^) and benzene ring (1590 and 1502 cm^−1^) (Figure S24). The ^1^H-NMR spectrum of **5** showed two sets of signals in the ratio of 1:0.75. Careful analysis of each set revealed that **5** is a mixture of two glycosides of **4** (Table 2, Figure S16). The anomeric proton signals (*δ*_H_ 5.68 (1H, d, *J* = 7.5 Hz, H-1″)/5.62 (1H, d, *J* = 7.5 Hz, H-1″)) and signals at *δ*_H_ 4.10–4.52 indicated the existence of the *β*-glucopyranosyl group in **5**. In the ^13^C- and DEPT NMR spectra, some signals also appeared as dual peaks (Table 2, Figure S17). Each peak was confirmed by analysis of the heteronuclear single quantum coherence (HSQC) and HMBC spectra (Figures S18 and S20). The HMBC spectrum of **5** showed the correlation between the anomeric proton (*δ*_H_ 5.68/5.62) and C-7 (*δ*_C_ 101.42/101.56), confirming the position of the *β*-glucopyranosyl group at C-7 (Figure 2 and Figure S20). On the basis of the above results, it was proposed that **5** is a mixture of C2 epimers. We tried to separate the epimers, but also failed. The absolute configuration of the sugar of **5** was determined as d-form by an acid hydrolysis and an HPLC experiment (Figure S26). Therefore, the planar structure of the new compound **5** was determined as 2-hydroxy-5-methoxy-naringenin 7-*O*-*β*-d-glucopyranoside. 

Compounds **2**–**3** and **6**–**22** were identified as 13-*N*-malonyl-l-tryptophan (**2**) [21], 13-*N*-caproyl-l-tryptophan-1-*N*-*β*-d-glucopyranoside (**3**) [5], 2-hydroxy-naringenin-5-*O*-*β*-d-glucopyranoside (**6**) [22], 2-hydroxy-eriodictyol-5-*O*-*β*-d-glucopyranoside (**7**) [23], apigenin (**8**) [24], nicotiflorin (**9**) [25], genistein (**10**) [26], tectorigenin (**11**) [27], irilin D (**12**) [28], tectoridin (**13**) [29], irisolidone-7-*O*-*β*-d-glucopyranoside (**14**) [30], tectorigenin-7-*O*-*β*-D-xylopyranosyl-(1→6)-*O*-*β*-d-glucopyronoside (**15**) [31], glycitein (**16**) [32], afromosin (**17**) [33], glycitin (**18**) [34], 7,4′-dihydroxy-6-methoxyisoflavone-7-*O*-*β*-d-xylopyranosyl-(1→6)-*O*-*β*-d-glucopyronoside (**19**) [31], gehuain (**20**) [35], (-)-hydnocarpin (**21**) [36], and hydrageifolin I (**22**) [37], by comparison with their NMR spectrum and previously published data. In previous studies, numerous isoflavonoids and saponins were isolated from *P*. *lobata* and puerarin; daidzein, tectoridin (**13**), and glycitin (**18**) were the most predominant constituents [5]. To the best of our knowledge, compounds **2**, **6**, **7**, **21**, and **22** were isolated from *P*. *lobata* for the first time in this work.

### 2.2. Cytotoxicity of Compounds Isolated from Puerariae Flos against Human Ovarian Cancer Cells

**Figure 3:**
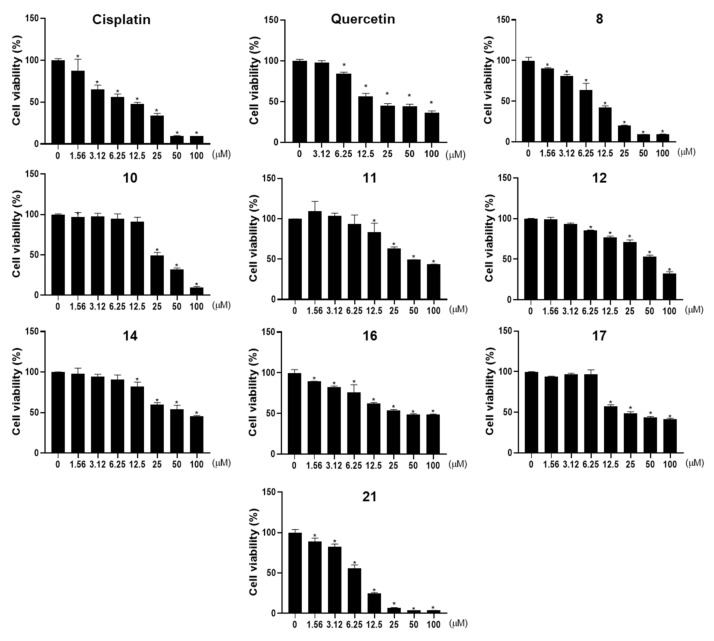
The cell viability of active compounds in A2780 human ovarian cancer cell line. Cisplatin was used as a positive control. The results are presented as mean standard ± SD and processed by using a one-way ANOVA. * *p* < 0.05 in comparison to control (0 μM).

To identify anti-tumor constituents in Pueraria Flos, we examined the effects of the 25 isolates on the cell viability of the human ovarian cancer A2780 cell line using the MTT assay. Among the isolated compounds, apigenin (**8**), genistein (**10**), tectorigenin (**11**), irilin D (**12**), irisolidone 7-*O*-glucoside (**14**), glycitein (**16**), afromosin (**17**), and (-)-hydnocarpin (**21**) showed cytotoxic activity (IC_50_ <100 μM) against A2780 cells (Figure 3, Table 3). Apigenin (**8**) and (-)-hydnocarpin (**21**) were the most active (IC_50_ values of 9.99 and 7.36 μM, respectively). In contrast, cisplatin, which is widely used as a first-line therapy in ovarian epithelial cancer, and quercetin, which is a well-known flavonoid to have anticancer activities, showed an IC_50_ value of 10.73 and 19. 45 μM, respectively. To further evaluate the effect of the compounds on the cell viability of non-malignant ovarian epithelial cells, we explored the cytotoxicity of the eight compounds (**8**, **10**, **11**, **12**, **14**, **16**, **17**, and **21**) against the immortalized human ovarian surface epithelial cell line IOSE80PC. (-)-Hydnocarpin (**21**) showed a mild cytotoxicity with an IC_50_ value of 65.84 μM against IOSE80PC cells and other seven compounds exhibited no activity (IC_50_ >100 μM) (Table 3). In contrast, cisplatin showed a similar cytotoxicity to IOSE80PC cells (IC_50_ value of 12.78 μM) as to A2780 cells (IC_50_ value of 10.73 μM). 

Previous studies demonstrate that apigenin (**8**) inhibits the proliferation and migration of ovarian cancer cell line A2780 by suppressing the expression of Id1 and focal adhesion kinase [38,39]. Genistein (**10**) inhibits cell proliferation and downregulates VEGF expression in the ovarian cancer cell line OVCAR3 [40]. Additionally, the anticancer activity of irilin D (**12**) and afromosin (**17**) in the breast cancer cell line MCF-7 has been reported [41,42]. This is the first report for the cytotoxic effect of irilin D (**12**), irisolidone 7-*O*-glucoside (**14**), afromosin (**17**), and (-)-hydnocarpin (**21**) on a human ovarian cancer cell line, to the best of our knowledge. Interestingly, (-)-hydnocarpin (**21**), a flavonolignan, exhibited the greatest effect on A2780 cells. (-)-Hydnocarpin (**21**) exhibited no cytotoxicity against multiple cancer cell lines (Lu1, LNCaP, and MCF-7) [43], but showed considerable antiproliferative activities against murine lymphocytic leukemia (P-388), kidney carcinoma (HEK293), and colon adenocarcinoma (SW-480) cell lines [44,45]. 

## 3. Materials and Methods

### 3.1. Plant Material

The flowers of *Pueraria lobata* (Willd.) Ohwi (Leguminoseae) were purchased from CK Pharm Co. (Seoul, Korea) in June 2019. The origin of the herbal material was identified by prof. Dae Sik Jang and a voucher specimen (PULO5-2019) has been stored in the Lab. of Natural Product Medicine, College of Pharmacy, Kyung Hee University, Seoul, Korea.

### 3.2. General Experimental Procedures

General experimental procedures are in the Supplementary Materials.

### 3.3. Extraction and Isolation

Dried flowers of *P. lobata* (1.6 kg) were extracted twice with methanol (16 L) at 80 °C for 2 h and the solvent was removed by rotary evaporator at 45 °C. The methanol extract (278.95 g) was fractionated by column chromatography (CC) using Diaion HP-20 with a gradient system of acetone and H_2_O (0:100 to 100:0 *v*/*v*) to give 17 fractions (F1~F17). 

F3 was separated by Sephadex LH-20 CC (5.6 × 59.0 cm) with 45% methanol to afford twelve fractions (F3-1~F3-12). Compound **22** (33.0 mg) was purified from F3-4 using medium pressure liquid chromatography (MPLC; Redi Sep-silica cartridge 80 g, CH_2_Cl_2_:methanol:H_2_O = 100:0:0 to 30:63:7, *v*/*v*/*v*). F3-7 was subjected to silica gel CC (230-400 mesh; 4.0 × 28.0 cm, CH_2_Cl_2_:methanol:H_2_O = 90:9:1 to 70:27:3 *v*/*v*/*v*) to obtain compounds **19** (342.8 mg) and **5** (44.7 mg). F3-10 was subjected to silica gel CC (230–400 mesh; 3.8 × 26.0 cm, CH_2_Cl_2_:methanol:H_2_O = 90:9:1 to 70:27:3 *v*/*v*/*v*) to give compounds **18** (24.4 mg), **6** (340.9 mg), and **7** (13.7 mg). F4 was fractionated further using Sephadex LH-20 CC (4.7 × 57.6 cm) with 50% methanol and produced six fractions (F4-1~F4-6). F4-5 yielded compound **6** (125.2 mg) by flash CC with Redi Sep-C18 cartridge (130 g, methanol:H_2_O, from 25:75 to 35:65 *v*/*v*). F5 was separated by Sephadex LH-20 CC (5.4 × 60.8 cm) with 50% methanol to produced nine fractions (F5-1~F5-9). F5-7 was subjected to MPLC with methanol and H_2_O mixture (from 30:70 to 35:65 *v*/*v*) to afford compound **6** (58.0 mg). F5-8 was subjected to silica gel CC (230–400 mesh; 3.8 × 26.2 cm, CH_2_Cl_2_:methanol:H_2_O = 90:9:1 to 65:26.5:3.5 *v*/*v*/*v*) to isolate compounds **13** (5.7 mg) and **18** (4.2 mg). F6 was fractionated further using Sephadex LH-20 CC (4.7 × 60.0 cm, 55% methanol) to give nine fractions (F6-1~F6-9). F6-5 was chromatographed over silica gel (230–400 mesh; 4.0 × 28.0 cm, CH_2_Cl_2_:methanol:H_2_O = 90:9:1 to 70:27:3 *v*/*v*/*v*) to obtain compounds **18** (2.8 mg) and **15** (205.1 mg). F6-7 yielded compound **2** (21.4 mg) by MPLC (40 g, CH_2_Cl_2_:methanol:H_2_O = 40:36:4 to 0:90:10 *v*/*v*/*v*). Compounds **4** (38.3 mg) and **9** (10.6 mg) were isolated from F6 to F8 by flash CC (CH_2_Cl_2_:methanol:H_2_O = 100:0:0 to 50:45:5 *v*/*v*/*v*). Compound **13** (3.5373 g) was obtained by recrystallization from F7 in methanol. The mother liquor was fractionated further using Sephadex LH-20 CC (4.7 × 66.5cm) to isolate compounds **4** (25.4 mg), **1** (20.1 mg), **20** (5.7 mg), and **18** (23.2 mg). F8 was fractionated by Sephadex LH-20 CC (5.6 × 52.5 cm, acetone) to obtain compound **3** (380.7 mg). F10 was subjected to Sephadex LH-20 CC (3.4 × 43.5 cm, 75% methanol) to obtain ten fractions (F10-1~F10-10). Compounds **14** (42.0 mg) and **12** (10.7 mg) were purified from F10-3 and F10-8, respectively, by MPLC. Compound **16** (16.6 mg) was recrystallized in F10-10 with methanol. F12 was separated by Sephadex LH-20 CC (5.4 × 68.0 cm, 75% methanol) to afford twelve fractions (F12-1~F12-12) and compounds **17** (16.6 mg) and **11** (1.54 g). F12-11 was chromatographed on silica gel (2.9 × 32.2 cm, CH_2_Cl_2_:methanol:H_2_O = 100:0:0 to 80:18:2 *v*/*v*/*v*) to isolate compound **10** (122.2 mg). Compounds **8** (13.8 mg), **16** (18.2 mg), and **21** (11.2 mg) were purified from F12-12 by silica gel CC (5.1 × 33.0 cm, CH_2_Cl_2_:methanol:H_2_O = 85:13.5:1.5 to 75:22.5:2.5 *v*/*v*/*v*).

#### 3.3.1. 13-*N*-Benzoyl-l-tryptophan-1-*N*-*β*-d-glucopyranoside (**1**)

Pale yellow powder; HR-ESI-Orbitrap-MS (positive mode) *m*/*z* = 471.1758 ([M+H]^+^; calculated for C_24_H_27_N_2_O_8_, 471.1762); ${\left[\alpha \right]}_{D}^{20}$: 33.6 (*c* 0.1, methanol); UV (methanol) λ_max_ nm (log ε): 204 (4.41), 223 (4.50), 272 (3.86); IR (ATR) ν_max_ 1718, 1630, 1522, 1460, 1322, 1222, 1071, 1015, and 712 cm^−1^; ^1^H- and ^13^C-NMR data, see Table 1.

#### 3.3.2. 2-Hydroxy-5-methoxy-naringenin (**4**)

Brown powder; HR-Q-TOF-MS (positive mode) *m*/*z* = 303.0879 ([M+H]^+^; calculated for C_16_H_14_O_6_, 303.0864); UV (methanol) λ_max_ nm (log ε): 215 (3.80), 286 (3.71), 324 (3.47); IR (ATR) ν_max_ 1676, 1588, and 1510 cm^−1^; ^1^H- and ^13^C-NMR data, see Table 2.

#### 3.3.3. 2-Hydroxy-5-methoxy-naringenin 7-*O*-*β*-d-glucopyranoside (**5**)

Brown powder; HR-ESI-Orbitrap-MS (positive mode) *m*/*z* = 465.1391 ([M+H]^+^; calculated for C_22_H_25_O_11_, 465.1392); UV (methanol) λ_max_ nm (log ε): 211 (4.43), 2.86 (4.26); IR (ATR) ν_max_ 1687 and 1590 cm^−1^; ^1^H- and ^13^C-NMR data, see Table 2.

### 3.4. Acidic Hydrolysis of Compounds ***1*** and ***5***

Compounds **1** and **5** (each 1.0 mg) were hydrolyzed with 2N HCl at 80 °C for 4 h and with 1N HCl at 100 °C for 3 h, respectively. Each reaction was stopped by the addition of sodium thiosulfate. Additional hydrolysis was proceeded for identifying the absolute configuration of tryptophan moiety of **1**. Compound **1** (1.0 mg) was hydrolyzed with 6N HCl at 85 °C for 24 h. Reaction was stopped by the addition of sodium thiosulfate.

### 3.5. Absolute Configuration Analysis of β-Glucoses in Compounds ***1*** and ***5***

To determine the absolute configuration of *β*-glucoses in **1** and **5**, the modified analysis method from a reference was conducted [46]. The hydrolysate was dissolved in pyridine (500 μL) and l-cysteine methyl ester hydrochloride (1.2 mg) was added and heated at 60 °C for 1 h. *σ*-Tolyl isothiocyanate (100 μL) was added and heated again at 60 °C for 1 h. The reaction product was analyzed by HPLC under a gradient system (A: 0.1 % (*v*/*v*) formic acid in water, B: 0.1 % (*v*/*v*) formic acid in acetonitrile, 10 to 50% B, 45 min). The glucoses in the reaction mixture of **1** and **5** were detected at 28.3 and 28.4 min each. Authentic l- and d-glucose were, respectively, detected at 27.6 and 28.2 min at the same HPLC conditions. Therefore, the absolute configuration of *β*-glucose in compounds **1** and **5** was identified as the d configuration.

### 3.6. Absolute Configuration Analysis of Tryptophan in Compound ***1***

The hydrolysate to determine the absolute configuration of tryptophan of 1 was subjected to HPLC by under a gradient system (A: water, B: methanol, 10 to 35% B, 80 min). Tryptophan (0.2 mg) was obtained from the hydrolysate. Analyzing the optical rotation dispersion of the obtained tryptophan revealed that it has l-configuration (${\left[\alpha \right]}_{D}^{22}$: −62.3, *c* 0.1, methanol) when compared to the optical rotation value of l-tryptophan (${\left[\alpha \right]}_{D}^{22}$: −74.0, *c* 0.1, methanol) and d-tryptophan (${\left[\alpha \right]}_{D}^{22}$: 103.8, *c* 0.1, methanol).

### 3.7. Cell Viability Assay

Human ovarian endometrioid adenocarcinoma cell line A2780 and immortalized ovarian surface epithelial cell line IOSE80PC were provided by Dr. Ie-Ming Shih (Johns Hopkins School of Medicine, Baltimore, MD, USA) and Dr. N. Auersperg (University of British Columbia, Vancouver, British Columbia, Canada), respectively. The cells were cultured in Roswell Park Memorial Institute (RPMI) 1640 supplemented with 5% fetal bovine serum (FBS), penicillin (100 U/mL), and streptomycin sulfate (100 μg/mL) in a 5% CO_2_ and 95% air humidified atmosphere at 37 °C. RPMI 1640, FBS, streptomycin sulfate, and penicillin were procured from Life Technologies Inc. (Grand Island, NY, USA). The cells were seeded at a density of 1.0 × 10^5^ cells/mL in a 96-well plate containing 50 μL of RPMI medium in each well and incubated for 24 h. Various concentrations of compounds dissolved in dimethyl sulfoxide (DMSO) were mixed with RPMI 1640 medium and added into cells in each well. The final concentration of DMSO in the medium did not exceed 0.1%. Following 48 h incubation, 50 μL of MTT (Molecular Probes Inc., Eugene, OR, USA) solution was added into each well to achieve a final concentration of 0.5 mg/mL and then incubated for an additional 4 h. The medium was discarded, and the formazan blue that formed in the cells was dissolved in 50 μL of DMSO. The optical density was measured at 540 nm by microplate spectrophotometer (SpectraMax; Molecular Devices, Sunnyvale, CA, USA). Three independent experiments with at least three replicates have been performed for all the tested compounds except quercetin, which has been tested once. IC_50_ is defined as the concentration that reduces cell number by 50% compared to control cultures. Results shown in Table 3 and Figure 3 are the representative of the independent experiments.

## 4. Conclusions

An investigation on compounds with cytotoxic activity in Puerariae Flos led to the isolation of 22 compounds (**1**–**22**), including one new tryptophan derivative, 13-*N*-benzoyl-l-tryptophan-1-*N-β*-d-glucopyranoside (**1**), and two new flavanones, 2-hydroxy-5-methoxy-naringenin (**4**) and 2-hydroxy-5-methoxy-naringenin 7-*O*-*β*-d-glucopyranoside (**5**). Apigenin (**8**), genistein (**10**), tectorigenin (**11**), irilin D (**12**), irisolidone 7-*O*-glucoside (**14**), glycitein (**16**), afromosin (**17**), and (-)-hydnocarpin (**21**) showed significant cytotoxicity against human ovarian cancer cell line A2780 and exhibited little cytotoxicity against human ovarian surface epithelial cell line IOSE80PC. (-)-Hydnocarpin (**21**), a flavonolignan, showed the most potent cytotoxic activity against A2780 cells.

## Figures and Tables

**Table 1:** ^1^H- and ^13^C-NMR spectroscopic data of compound **1** (*δ* in ppm, methanol-*d_4_*, 500 MHz, and 125 MHz).

Position *^a^*	1	
	*δ_C_*	*δ*_H_ *^b^*
1		
2	125.5	7.27 s
3	112.7	
4	120.0	7.52 d (8.0)
5	121.1	6.92 ddd (8.0, 7.0, 1.0)
6	123.2	7.10 ddd (8.0, 7.0, 1.0)
7	111.8	7.47 d (7.0)
8	130.4	
9	138.5	
10	28.3	3.34 overlapped/3.49 overlapped
11	55.6	4.78 t (5.0)
12	175.9	
13		
Glc-1′	86.8	5.41 d (9.0)
Glc-2′	73.8	3.92 t (9.0)
Glc-3′	79.1	3.60 t (9.0)
Glc-4′	71.6	3.49 overlapped
Glc-5′	80.7	3.55 ddd (10.0, 6.0, 2.5)
Glc-6′	62.9	3.67 dd (12.0, 6.0)/3.86 dd (12.0, 2.0)
1″	135.6	
2″/6″	129.7	7.67 dd (8.5, 1.5)
3″/5″	128.6	7.38 dd (8.5, 7.5)
4″	132.9	7.46 tt (7.5, 1.5)
7″	170.4	

***^a^*** All assignments were based on COSY, HSQC, and HMBC results. ***^b^*** *δ*_H_ Multi (*J* in Hz).

**Table 2:** ^1^H- and ^13^C-NMR spectroscopic data of compounds **4** and **5** (*δ* in ppm, pyridine-*d*_5_, 500 MHz, and 125 MHz).

Position *^a^*	4		5	
	*δ_C_*	δ_H_^*b*^	δ_C_	*δ*_H_ *^b^*
2	107.6		107.20	
3	42.4	3.66 q (14.0)	41.72	3.64 overlapped
4	194.3		195.08	
5	160.6		159.37/159.45	
6	94.8	6.12 d (1.5)	93.84/82.78	6.14 d (2.0)/6.25 d (2.0)
7	173.5		101.42/101.56	
8	92.7	6.49 d (1.5)	92.20/92.58	6.81 d (2.0)/6.76 d (2.0)
9	174.2		173.34/173.19	
10	102.1		168.40/168.46	
1′	126.1		125.11	
2′/6′	132.9	7.57 d (8.5)	132.38	7.54 d (8.5)/7.51 d (8.5)
3′/5′	116.2	7.05 d (8.5)	115.71/115.68	7.06 d (8.5)/7.04 d (8.5)
4′	158.1		157.68	
Glc-1″			104.48/104.53	5.68 d (7.5)/5.62 d (7.5)
Glc-2″			74.57	4.34 overlapped
Glc-3″			78.25	4.34 overlapped
Glc-4″			70.85/70.92	4.34 overlapped
Glc-5″			78.91/78.98	4.10 m
Glc-6″			61.96/62.01	4.34 overlapped/4.52 m
3′-OMe	55.7	3.56 s	55.48/55.54	3.55 s/3.60 s

***^a^*** All assignments were based on COSY, HSQC, and HMBC results. ***^b^*** *δ*_H_ Multi (*J* in Hz).

**Table 3:** The cytotoxicity of compounds **1**−**22** isolated from Pueraria Flos against human ovarian cell lines A2780 and IOSE80PC.

Compound	IC_50_ (μM) *^a^*	
	A2780	IOSE80PC
**8**	9.99 ± 0.98	>100
**10**	25.63 ± 1.80	>100
**11**	48.67 ± 0.31	>100
**12**	57.45 ± 3.08	>100
**14**	71.24 ± 11.29	>100
**16**	48.54 ± 1.97	>100
**17**	23.72 ± 2.54	>100
**21**	7.36 ± 0.58	65.84 ± 1.60
Cisplatin	10.73 ± 0.81	12.78 ± 0.55
Quercetin	19.45 ± 2.92	>100

*^a^* IC_50_ value is defined as the concentration that reduces cell number by 50% compared to control cultures. Compounds **1**–**7**, **9**, **13**, **15**, **18**–**20**, and **22** were not active (IC_50_ > 100 μM) in A2780 cells. The inactive compounds were not tested on IOSE80PC.

## Data Availability

Not applicable.

## Supplementary Materials

The following supporting information can be downloaded at: https://www.mdpi.com/article/10.3390/plants11131651/s1, General experimental procedure; The HR-ESI-ORBITRAP-MS, IR, ^1^H-NMR (500 MHz, methanol-*d*_4_), ^13^C-NMR (125 MHz, methanol-*d*_4_), HSQC, COSY, HMBC, NOESY spectra of **1** (Figures S1–S7 and S22), The HR-Q-TOF-MS, IR, ^1^H-NMR (500 MHz, pyridine-*d*_5_), ^1^H-NMR (500 MHz, DMSO-*d*_6_), ^13^C-NMR (125 MHz, pyridine-*d*_5_), HSQC, COSY, HMBC, NOESY spectra of **4** (Figures S8–S14 and S23), The HR-ESI-ORBITRAP-MS, IR, ^1^H-NMR (500 MHz, pyridine-*d*_5_), ^13^C-NMR (125 MHz, pyridine-*d*_5_), HSQC, COSY, HMBC, NOESY spectra of **5** (Figures S15–S21 and S24), Analysis of sugar by HPLC (A) hydrolyzed sugar part of compound **1**; (B) d-glucose; (C) l-glucose (Figure S25), Analysis of sugar by HPLC (A) hydrolyzed sugar part of compound **5**; (B) d-glucose; (C) l-glucose (Figure S26).
