# Supplementary material for: Chemical Constituents of the Flowers of Pueraria lobata and Their Cytotoxic Properties
Source: Plants (Basel). 2022 Jun 22;11(13):1651. doi: 10.3390/plants11131651 (PMC9269118; doi:10.3390/plants11131651)

plants

MDPI

Supplementary materials

Chemical Constituents of the Flowers of *Pueraria lobata* and their cytotoxic properties.

Contents

General Experimental procedure
Figure S1. The HR-ESI-ORBITRAP-MS spectrum of compound **1**
Figure S2. The ^{1}H-NMR (500 MHz, methanol-d_4) spectrum of compound **1**
Figure S3. The ^{13}C-NMR (125 MHz, methanol-d_4) spectrum of compound **1**
Figure S4. The HSQC spectrum of compound **1** in methanol-d_4
Figure S5. The COSY spectrum of compound **1** in methanol-d_4
Figure S6. The HMBC spectrum of compound **1** in methanol-d_4
Figure S7. The NOESY spectrum of compound **1** in methanol-d_4
Figure S8. The HR-Q-TOF-MS spectrum of compound **4**
Figure S9. The ^{1}H-NMR (500 MHz, pyridine-d_5) spectrum of compound **4**
Figure S9a. The The ^{1}H-NMR (500 MHz, DMSO-d_6) spectrum of compound **4**
Figure S10. The ^{13}C-NMR (125 MHz, pyridine-d_5) spectrum of compound **4**
Figure S11. The HSQC spectrum of compound **4** in pyridine-d_5
Figure S12. The COSY spectrum of compound **4** in pyridine-d_5
Figure S13. The HMBC spectrum of compound **4** in pyridine-d_5
Figure S14. The NOESY spectrum of compound 1 in pyridine-d_5
Figure S15. The HR-ESI-ORBITRAP-MS spectrum of compound **5**
Figure S16. The ^{1}H-NMR (500 MHz, pyridine-d_5) spectrum of compound **5**
Figure S17. The ^{13}C-NMR (125 MHz, pyridine-d_5) spectrum of compound **5**
Figure S18. The HSQC spectrum of compound **5** in pyridine-d_5
Figure S19. The COSY spectrum of compound **5** in pyridine-d_5
Figure S20. The HMBC spectrum of compound **5** in pyridine-d_5
Figure S21. The NOESY spectrum of compound **5** in pyridine-d_5
Figure S22. The IR spectrum of compound **1**
Figure S23. The IR spectrum of compound **4**
Figure S24. The IR spectrum of compound **5**

Figure S25. Analysis of sugar by HPLC (A) hydrolyzed sugar part of compound **1**; (B) D-glucose; (C) L-glucose.
Figure S26. Analysis of sugar by HPLC (A) hydrolyzed sugar part of compound **5**; (B) D-glucose; (C) L-glucose.

General Experimental procedure

UV spectra were evaluated on Optizen pop (Mecasys, Daejeon, Korea). Optical rotations were obtained on a Jasco P-2000 polarimeter (JASCO, Tokyo, Japan), using a 1 cm microcell. JEOL 500 MHz NMR (JEOL, Tokyo, Japan) was used for obtaining NMR spectra. HR-Mass spectra were obtained by a Q-TOF micro mass spectrometer (Agilent Technologies, Santa Clara, CA, USA) and a hybrid orbitrap mass spectrometer (Q-Exative Focus, Thermo Fisher Sientific, San Jose, CA, USA). TLC analyses were performed on Silica gel 60 F_{254} (Merck, Kenilworth, MA, USA) and RP-18 F_{254S} (Merck) plates. Compounds were visualized by dipping plates into 20% (v/v) H_2SO_4 reagent (Samchun) and then heated at 110°C for 5-10 min. Agilent Cary 630 FTIR (Agilent Technologies, Santa Clara, CA, USA) was applied to obtain IR spectrum. Sephadex LH-20 (Amersham Pharmacia Biotech, Buckinghamshire, United Kingdom), Silica gel (Merck 60A, 230-400 mesh ASTM), Diaion HP-20 (Mitsubishi, Tokyo, Japan), and reversed-phase silica gel (YMC Co., ODS-A 12 nm S-150 μm) were used for column chromatography. Pre-packed cartridges, Redi Sep-Silica (12 g, 24 g, 40 g, Teledyne Isco) Combiflash PF, with Redi Sep-Silica (12 g, 24 g, 40 g, Teledyne Isco) and Redi Sep-C18 (13 g, 26 g, 43 g, 130 g, Teledyne Isco) were used for flash chromatography. HPLC was performed using Waters purification system (1525 pump, PDA 1996 detector) with Gemini NX-C18 110A column (250 × 21.2mm i.d. 5 μm, Phenomenex, Torrance, CA, USA). Flash chromatography was performed using the flash purification system (Combi Flash Rf, Teledyne Isco). Before chromatographic separations, all solvents used for this study were distilled.

Figure S1. The HR-ESI-ORBITRAP-MS spectrum of compound **1**

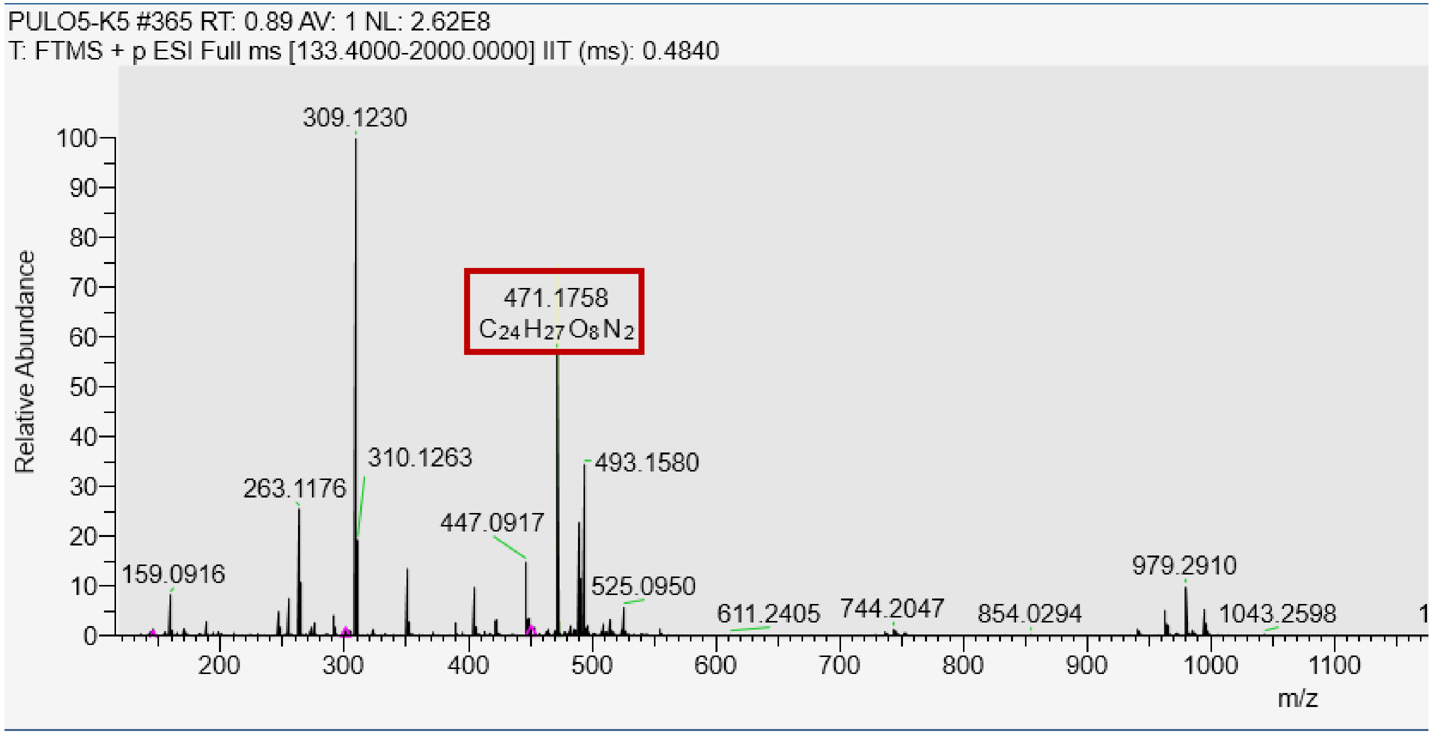

Elemental Composition Results - Spectrum 2

Peak Mass	Display Formula	S Fit	RDB	Delta [ppm]	Theo. mass	Rank
471.1758	$C_{24}H_{27}O_8N_2$	64.59	12.50	-0.74	471.17619	1
471.1758	$C_8H_{27}O_{13}N_{10}$	13.09	0.50	1.03	471.17536	4
471.1758	$C_{25}H_{23}O_4N_6$	33.65	17.50	-3.58	471.17753	2
471.1758	$C_{12}H_{31}O_{15}N_4$	20.71	-0.50	-4.67	471.17804	3
471.1758	$C_{20}H_{23}O_6N_8$	6.57	13.50	4.96	471.17351	5

Figure S2. The ^{1}H-NMR (500 MHz, methanol-d_4) spectrum of compound **1**

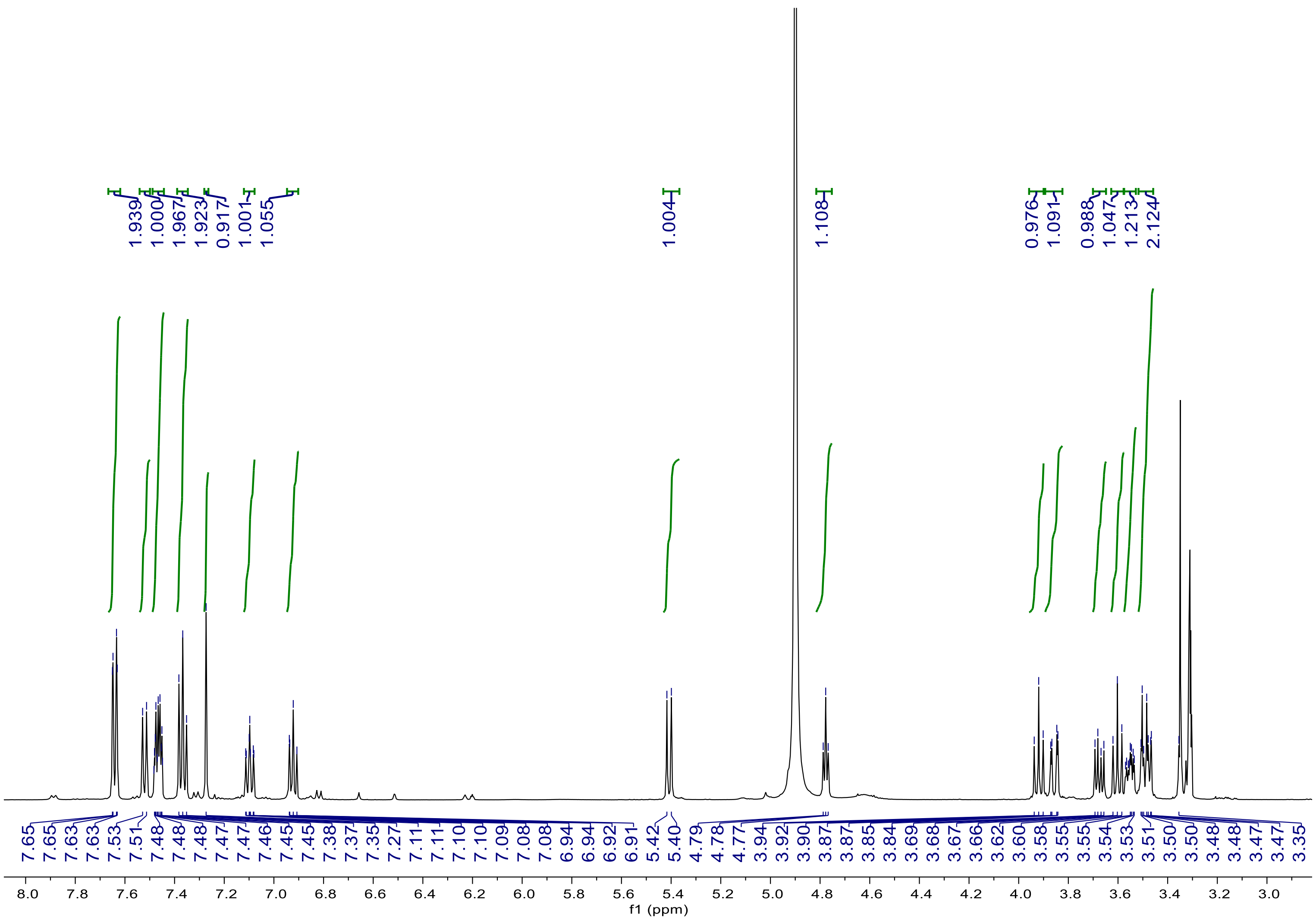

Figure S3. The ^{13}C-NMR (125 MHz, methanol-d_4) spectrum of compound **1**

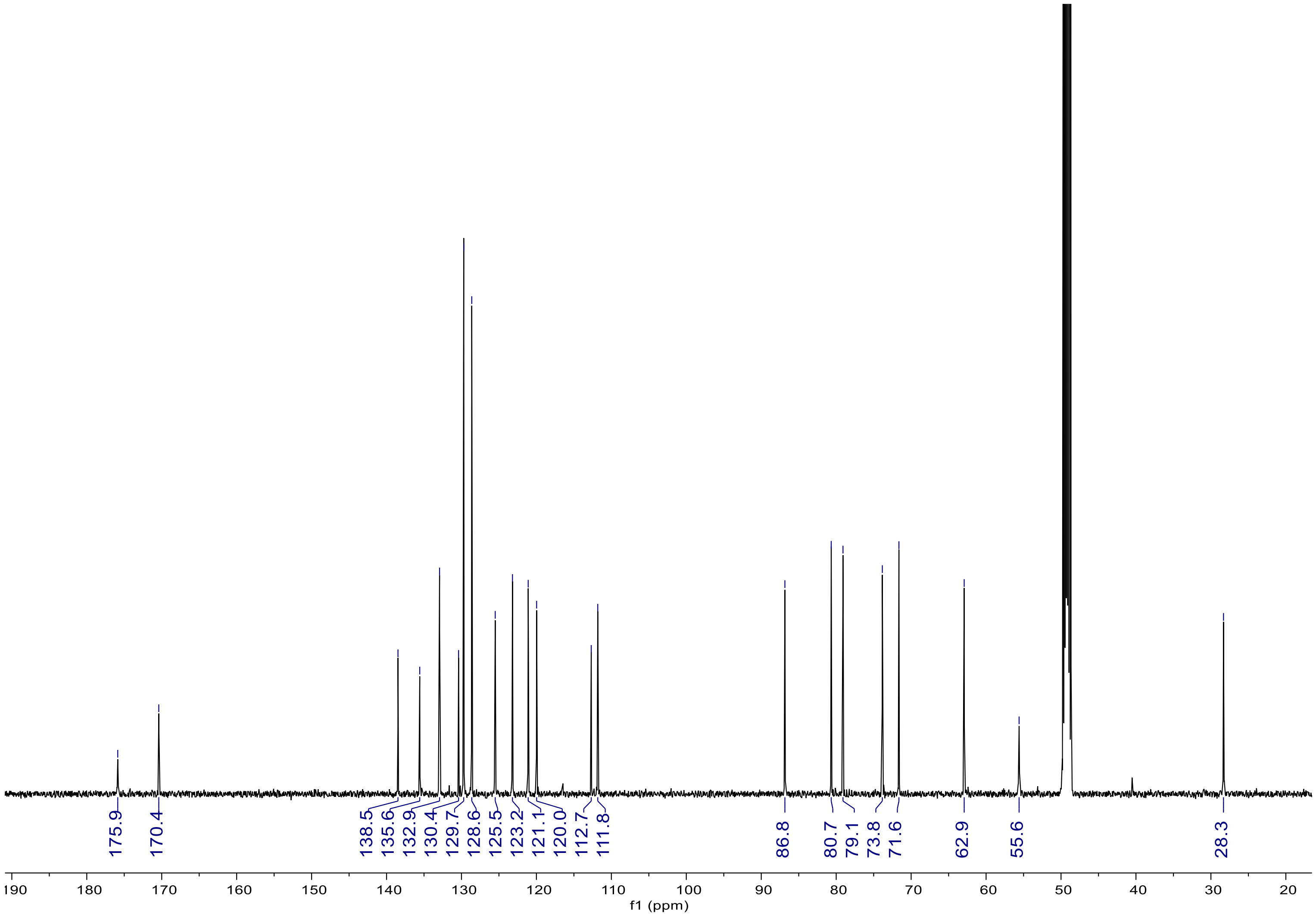

Figure S4. The HSQC spectrum of compound **1** in methanol-d_4

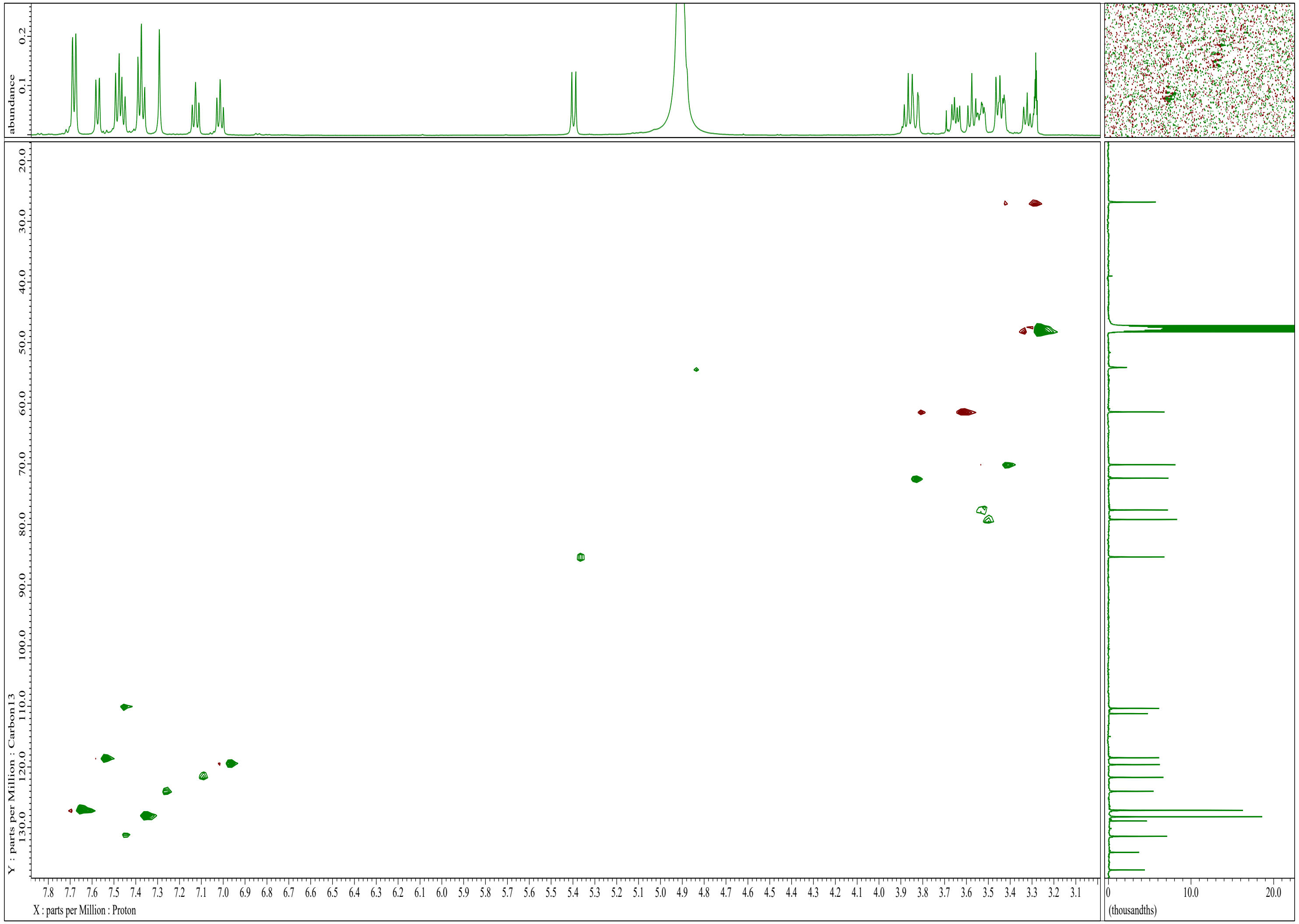

Figure S5. The COSY spectrum of compound **1** in methanol-d_4

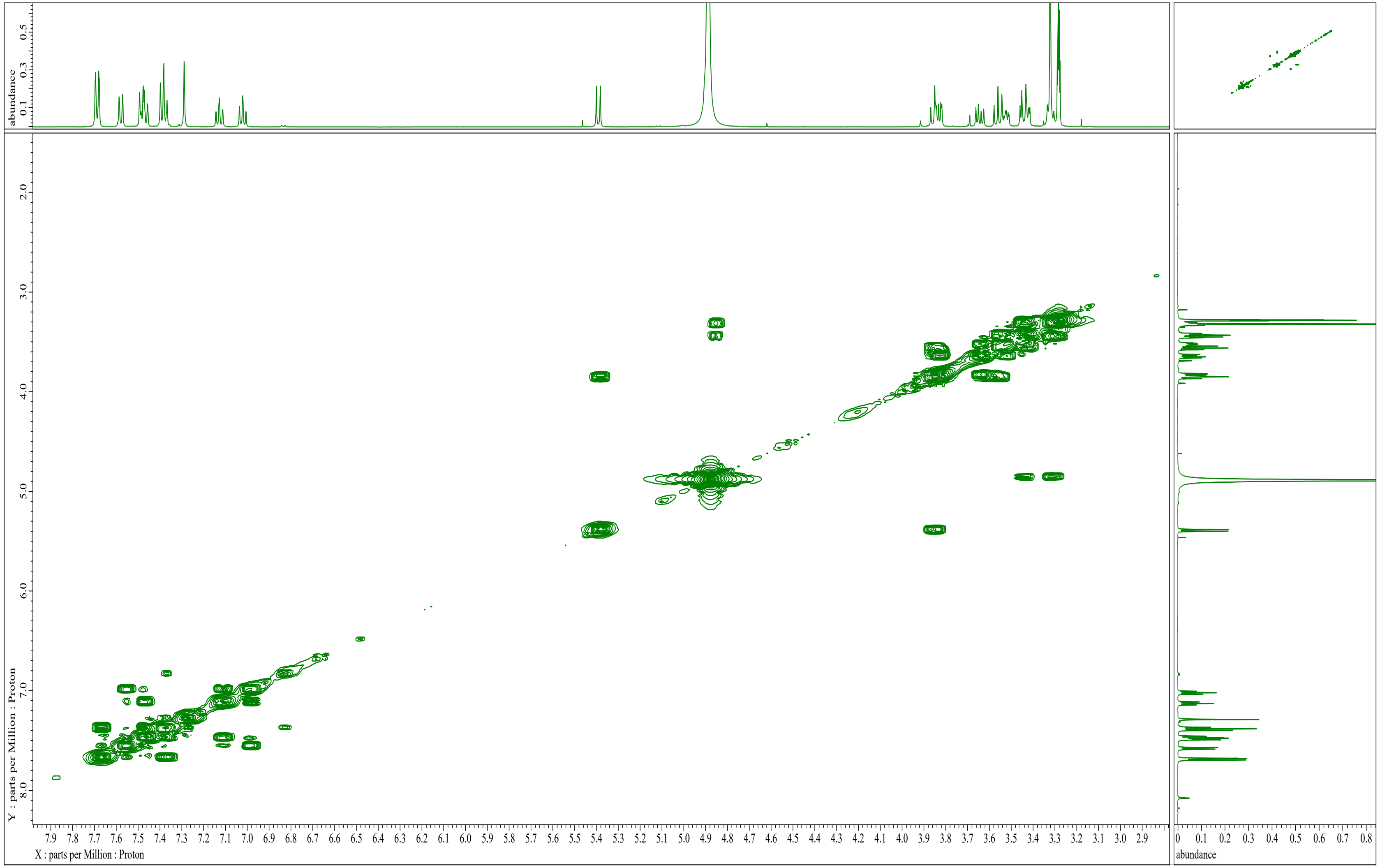

Figure S6. The HMBC spectrum of compound **1** in methanol-d_4

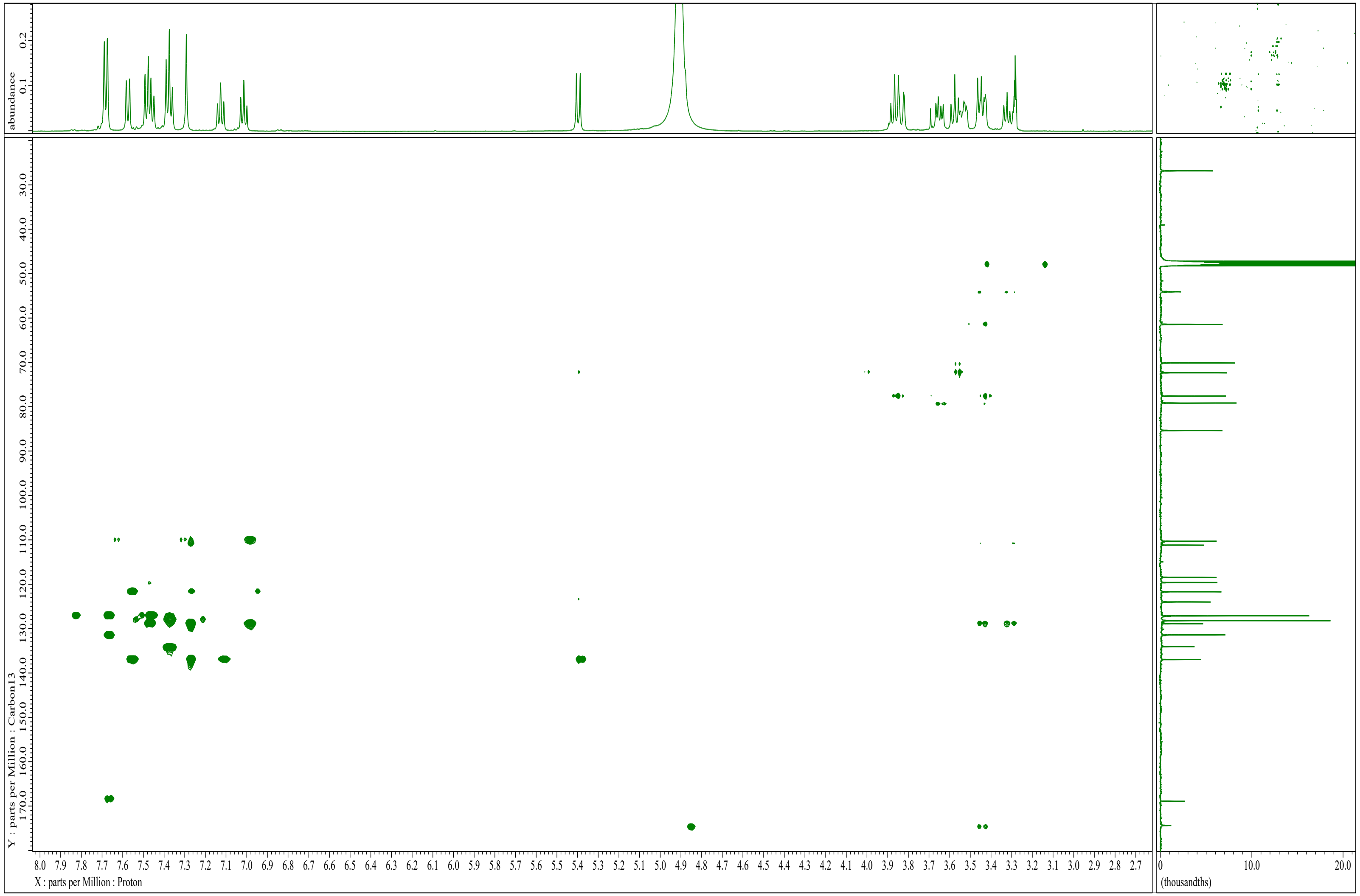

Figure S7. The NOESY spectrum of compound 1 in methanol-d_4

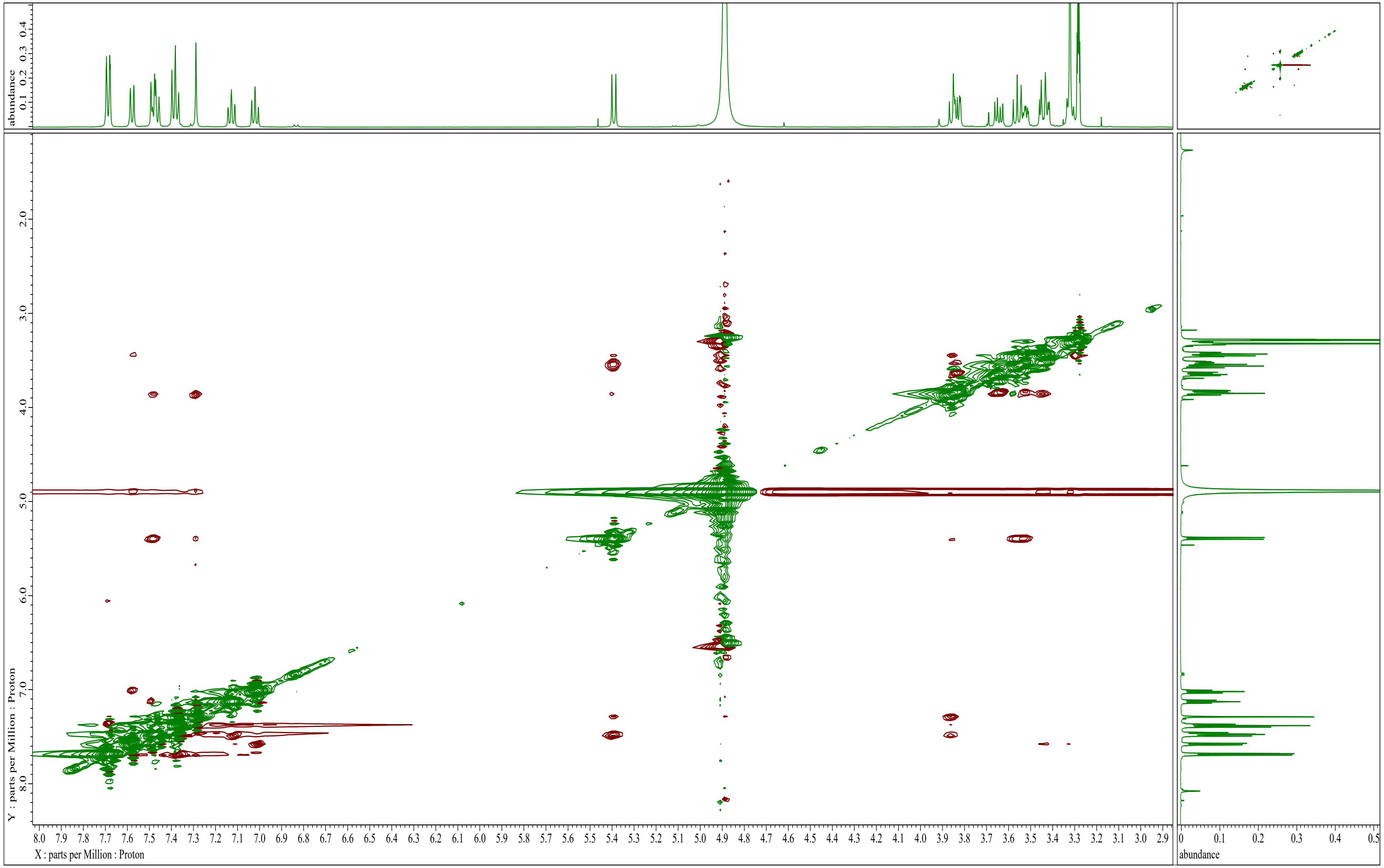

Figure S8. The HR-Q-TOF-MS spectrum of compound **4**

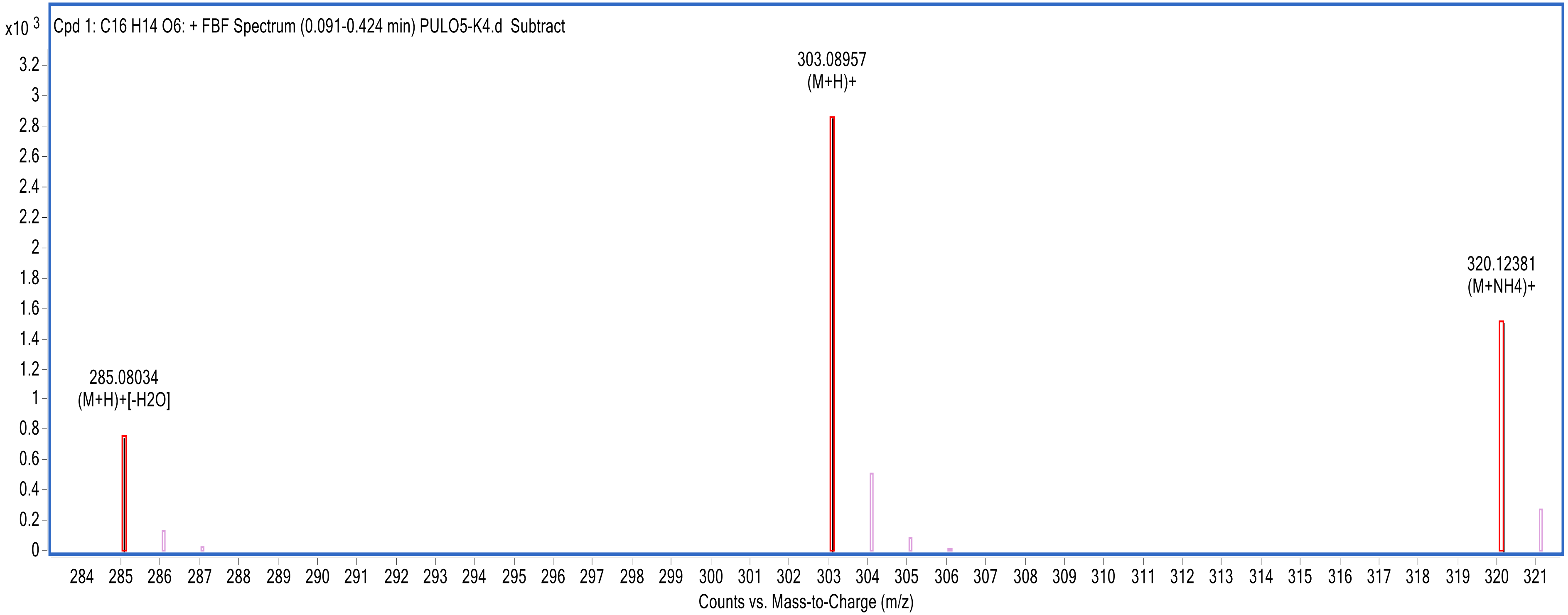

Figure S9. The ^{1}H-NMR (500 MHz, pyridine-d_5) spectrum of compound **4**

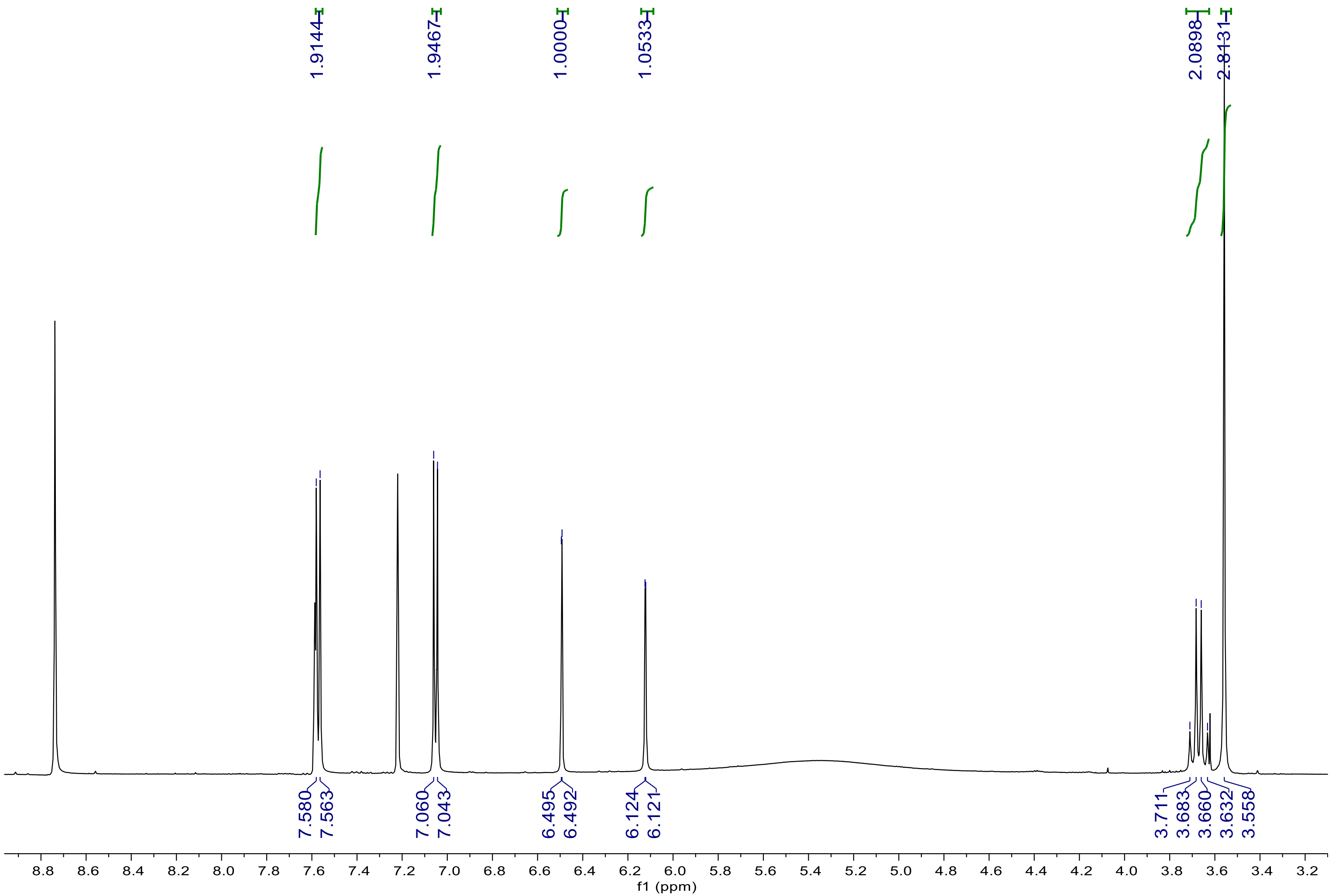

Figure S9a. The The ^{1}H-NMR (500 MHz, DMSO-d_6) spectrum of compound **4**

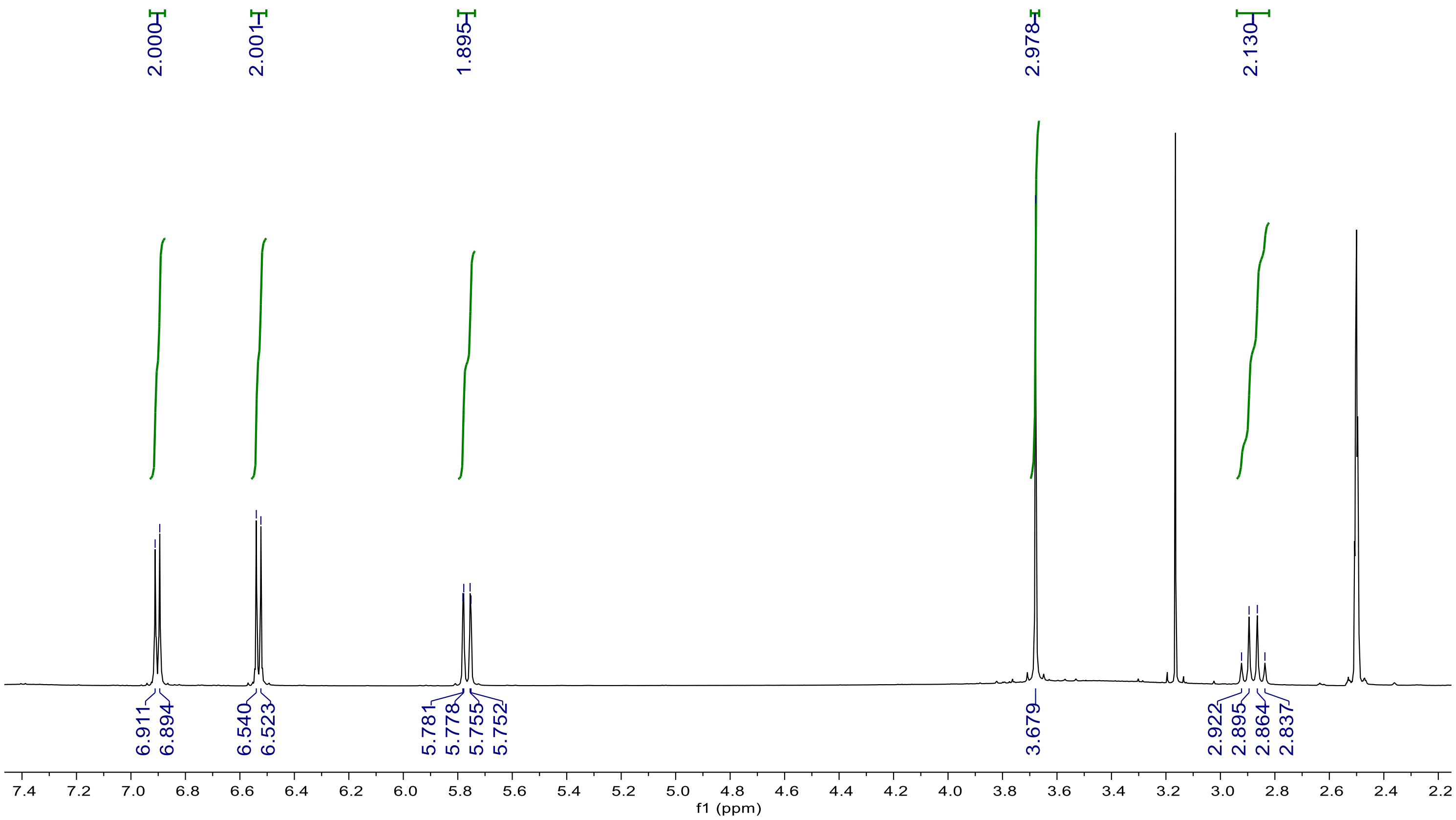

Figure S10. The ^{13}C-NMR (125 MHz, pyridine-d_5) spectrum of compound **4**

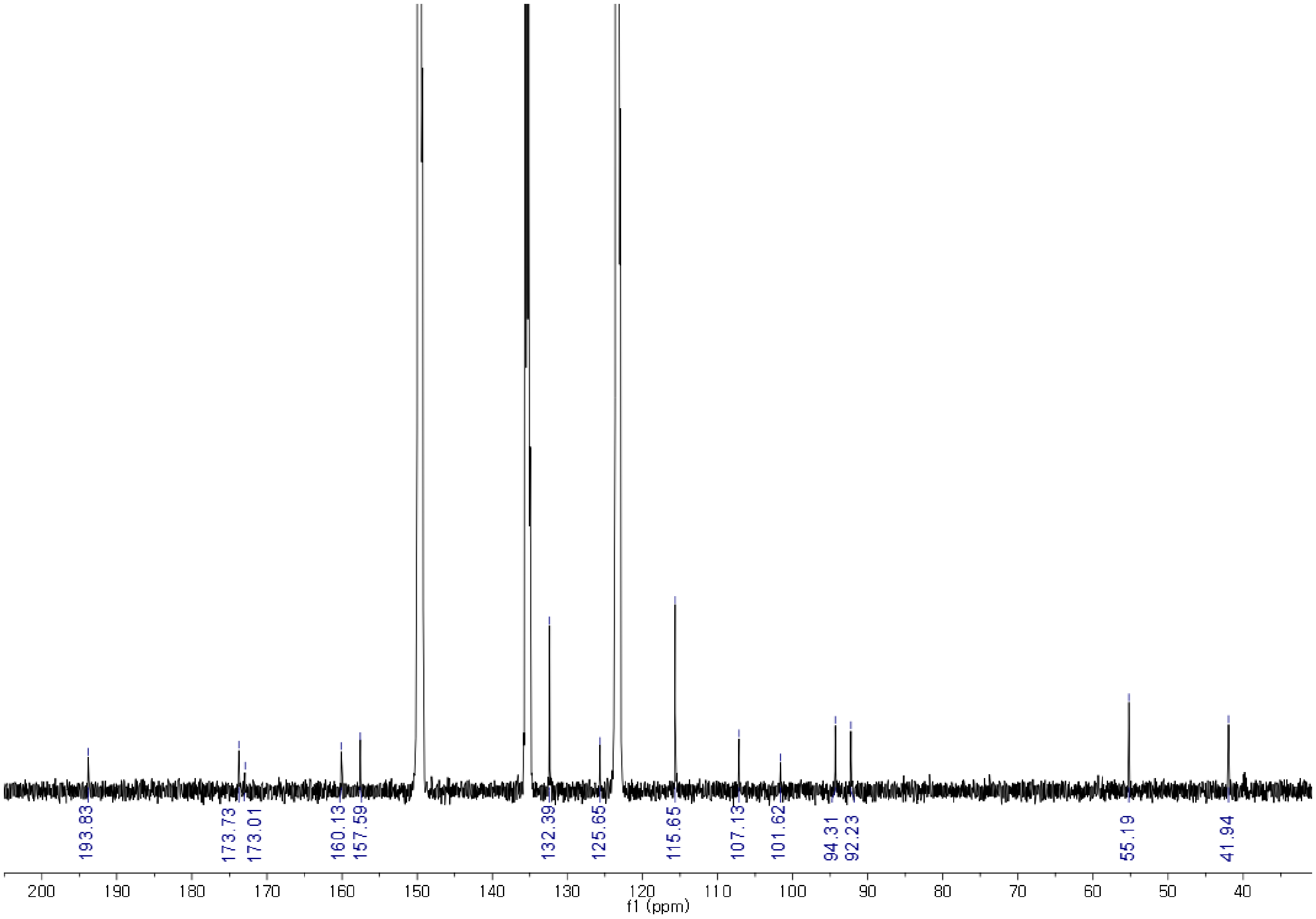

Figure S11. The HSQC spectrum of compound **4** in pyridine-*d*5

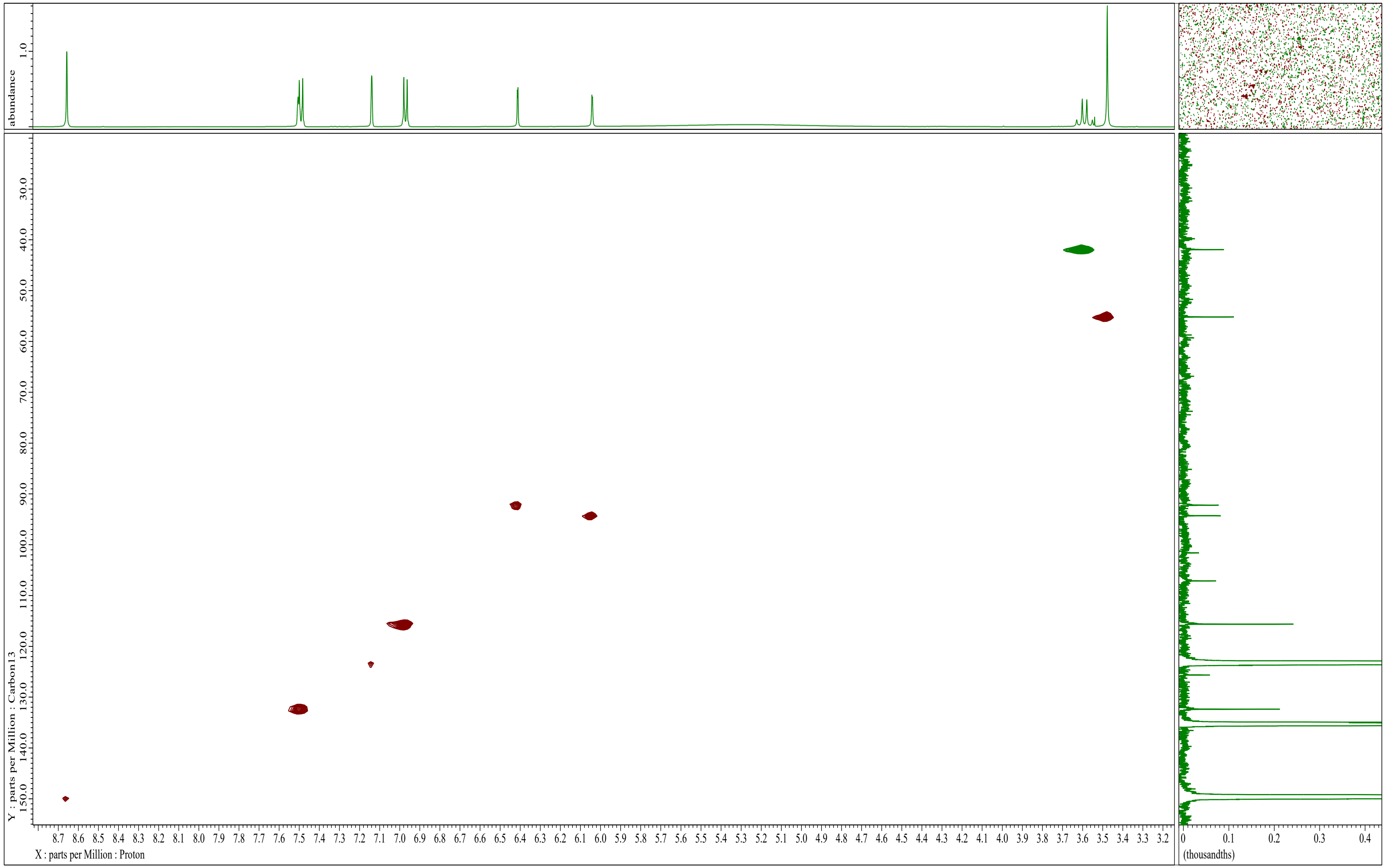

Figure S12. The COSY spectrum of compound **4** in pyridine-d_5

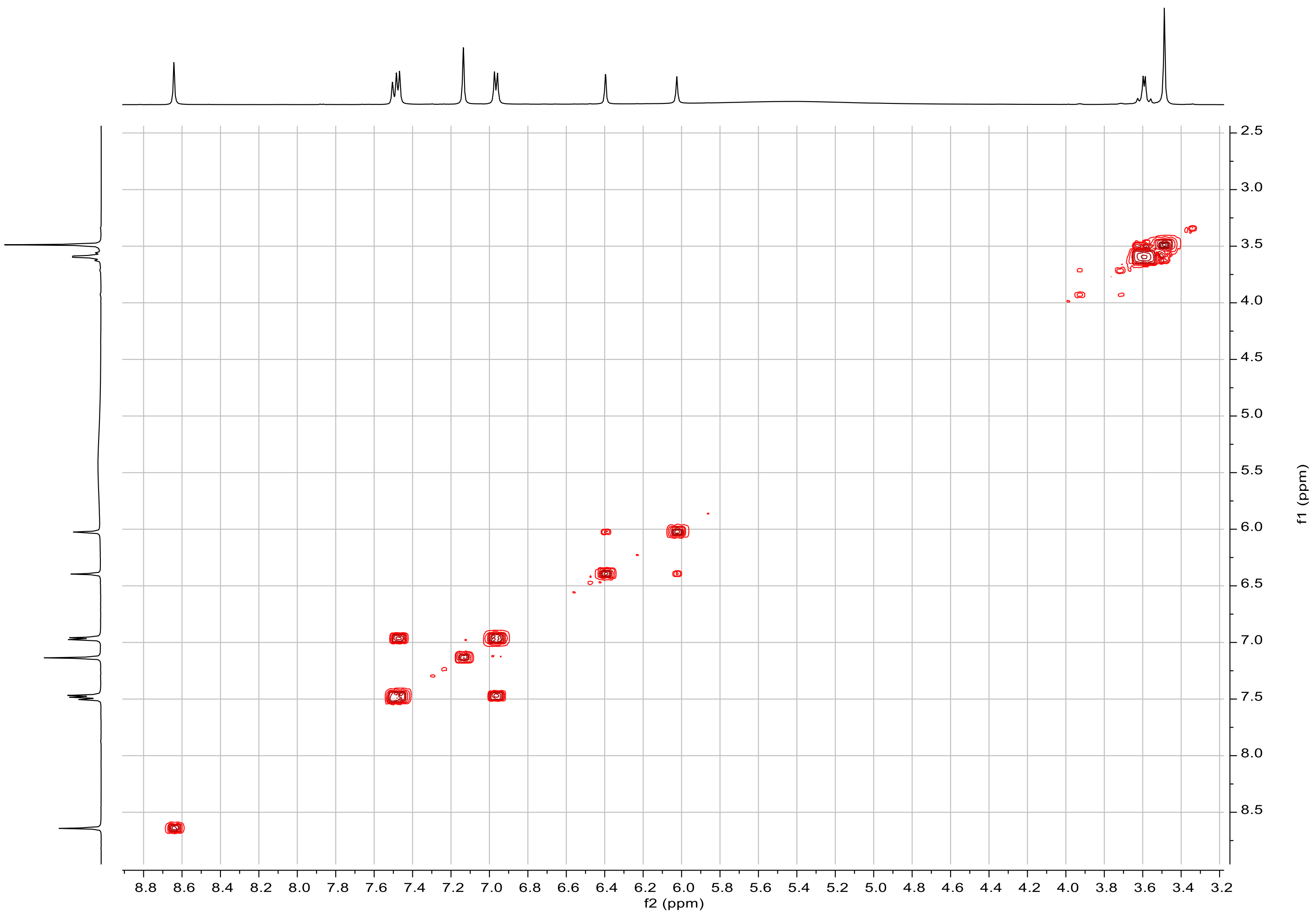

Figure S13. The HMBC spectrum of compound **4** in pyridine-*d*5

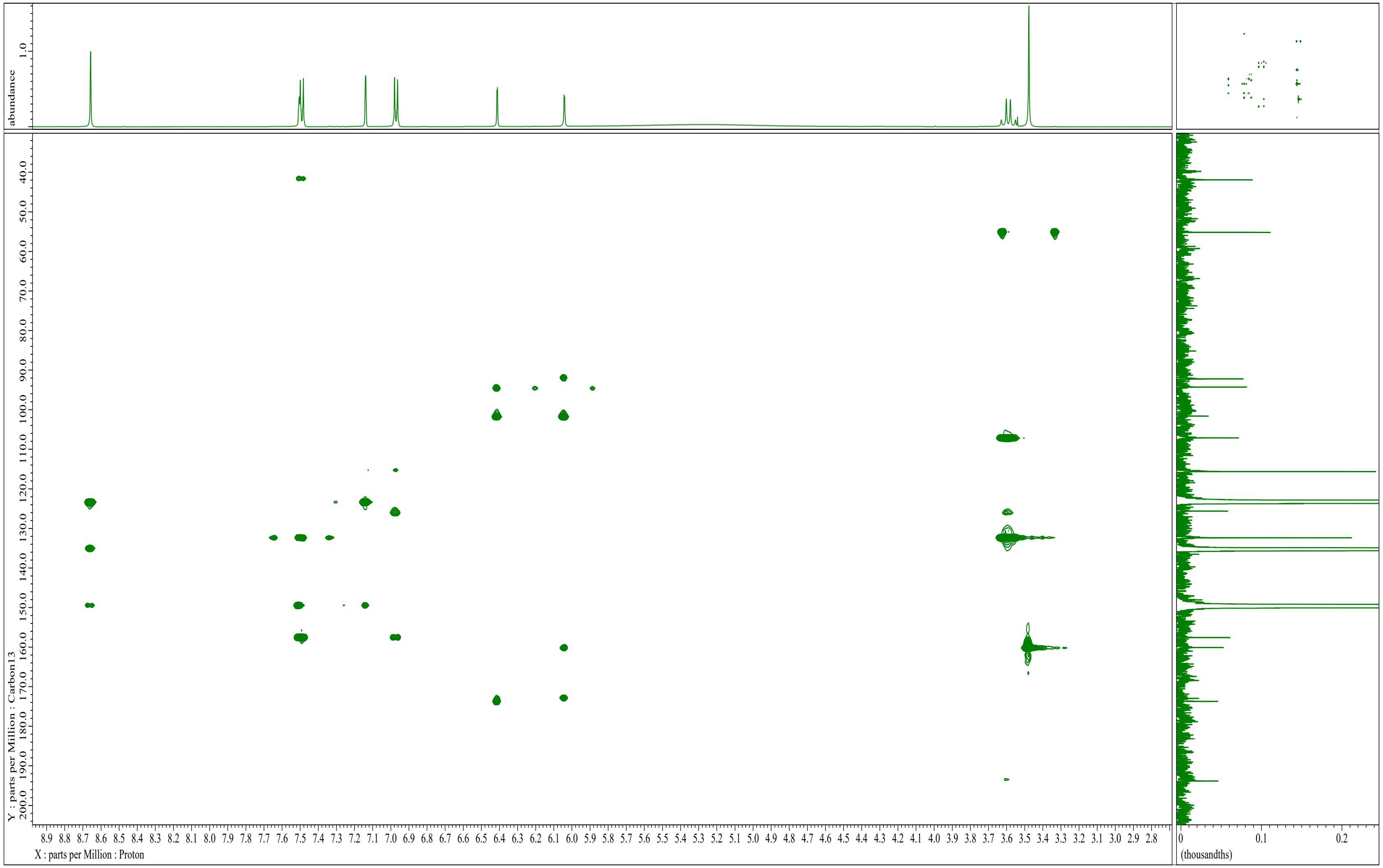

Figure S14. The NOESY spectrum of compound 1 in pyridine-*d*5

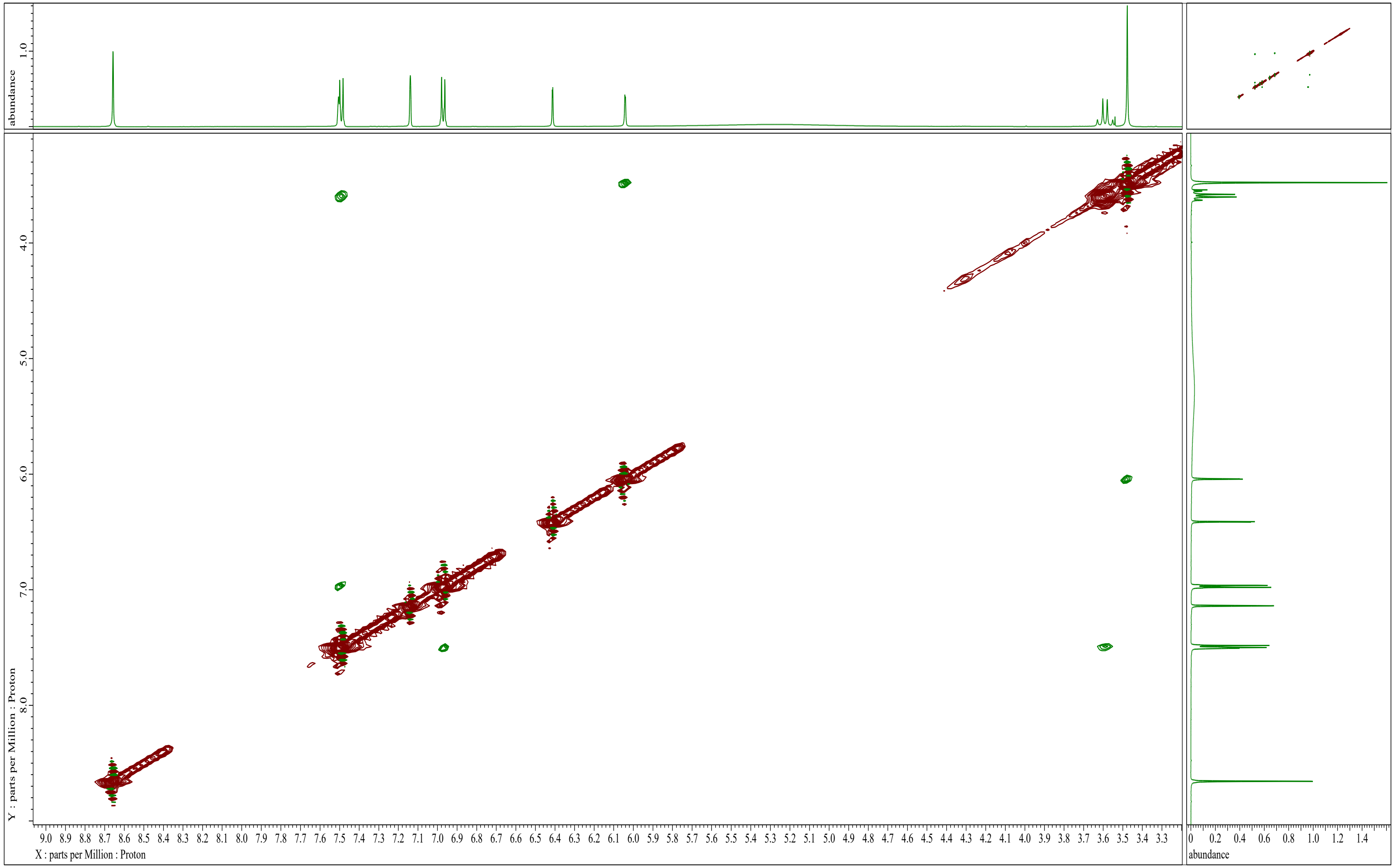

Figure S15. The HR-ESI-ORBITRAP-MS spectrum of compound **5**

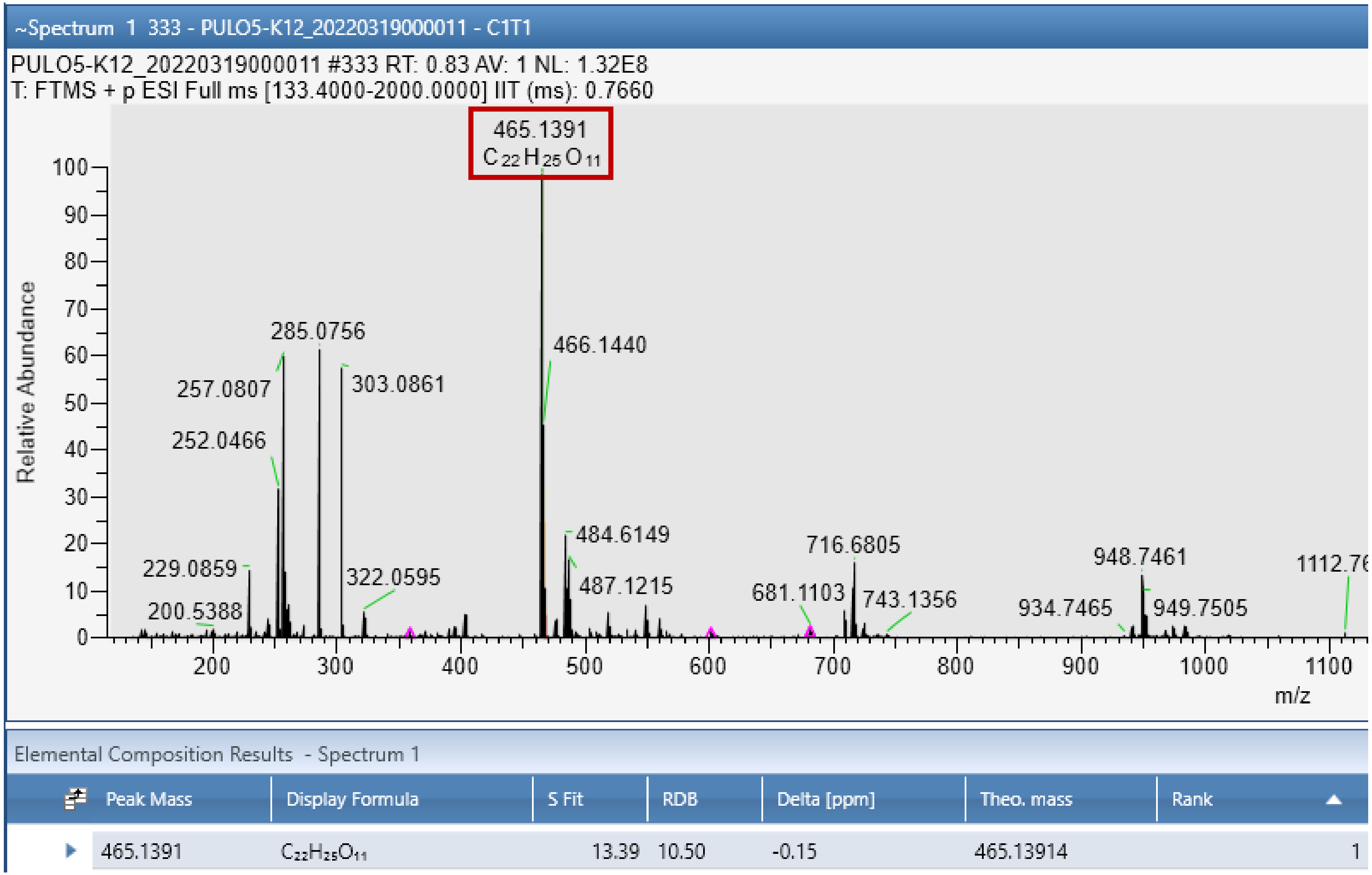

Figure S16. The ^{1}H-NMR (500 MHz, pyridine-d_5) spectrum of compound **5**

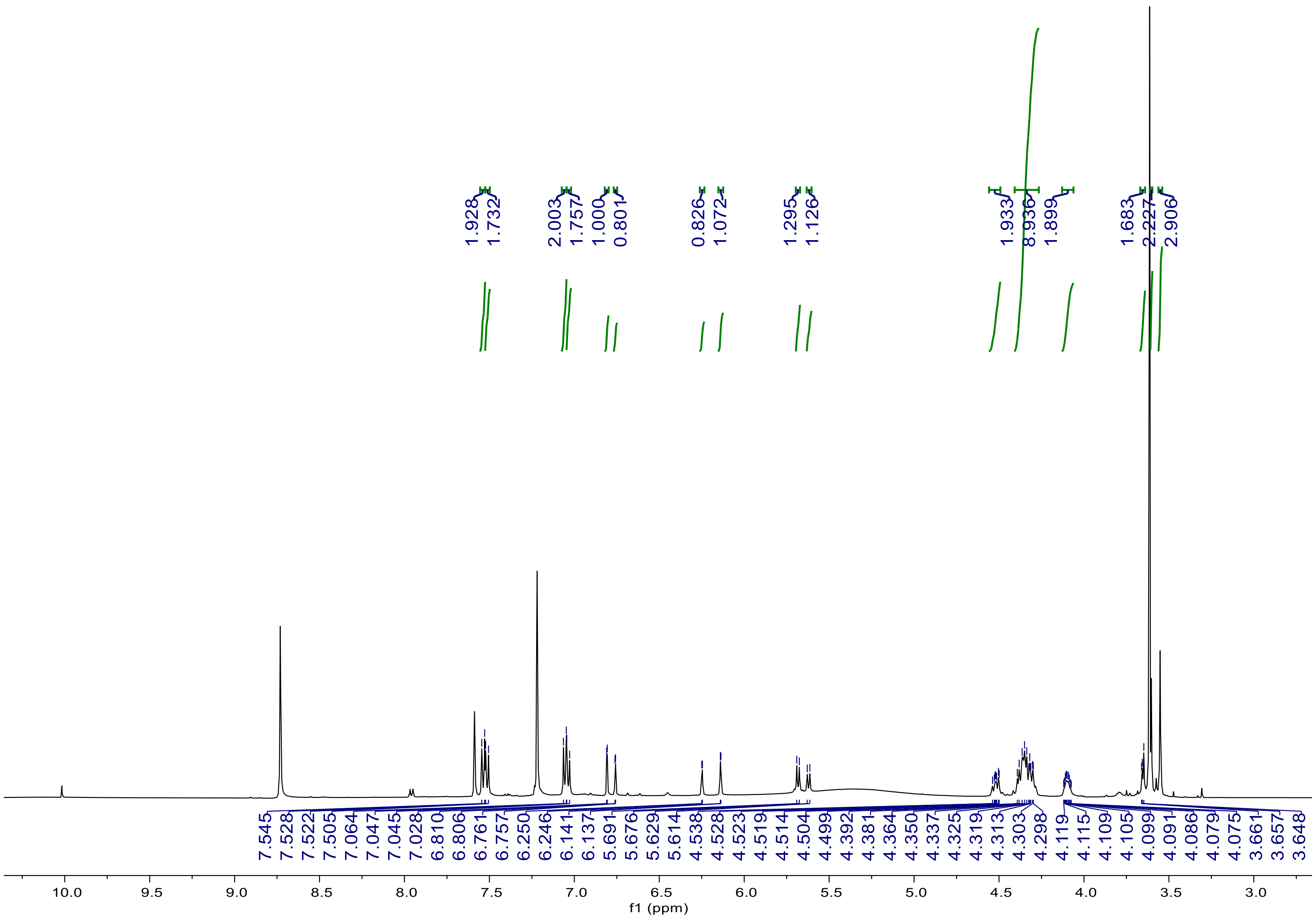

Figure S17. The ^{13}C-NMR (125 MHz, pyridine-*d*$_5$) spectrum of compound **5**

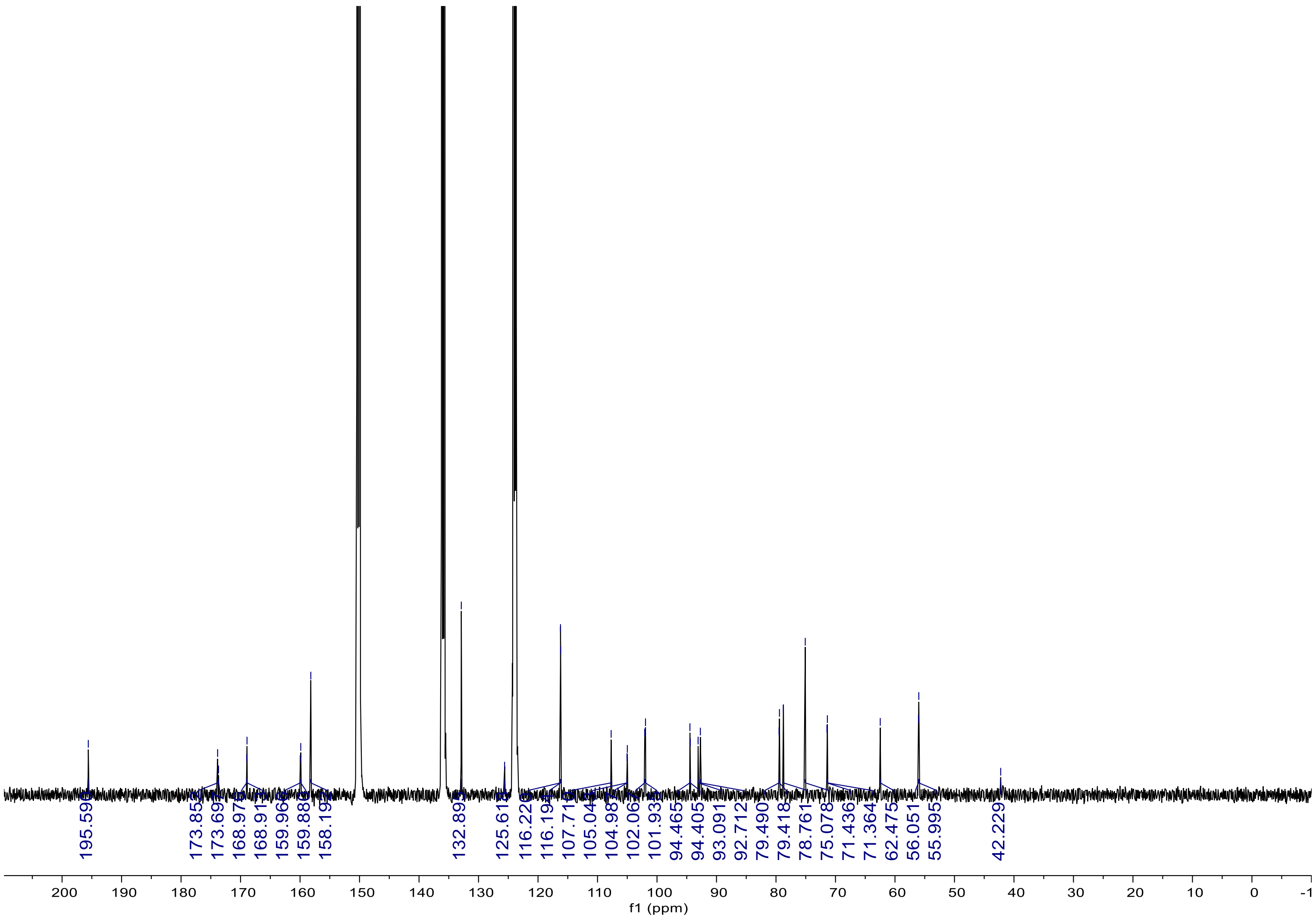

Figure S18. The HSQC spectrum of compound **5** in pyridine-d_5

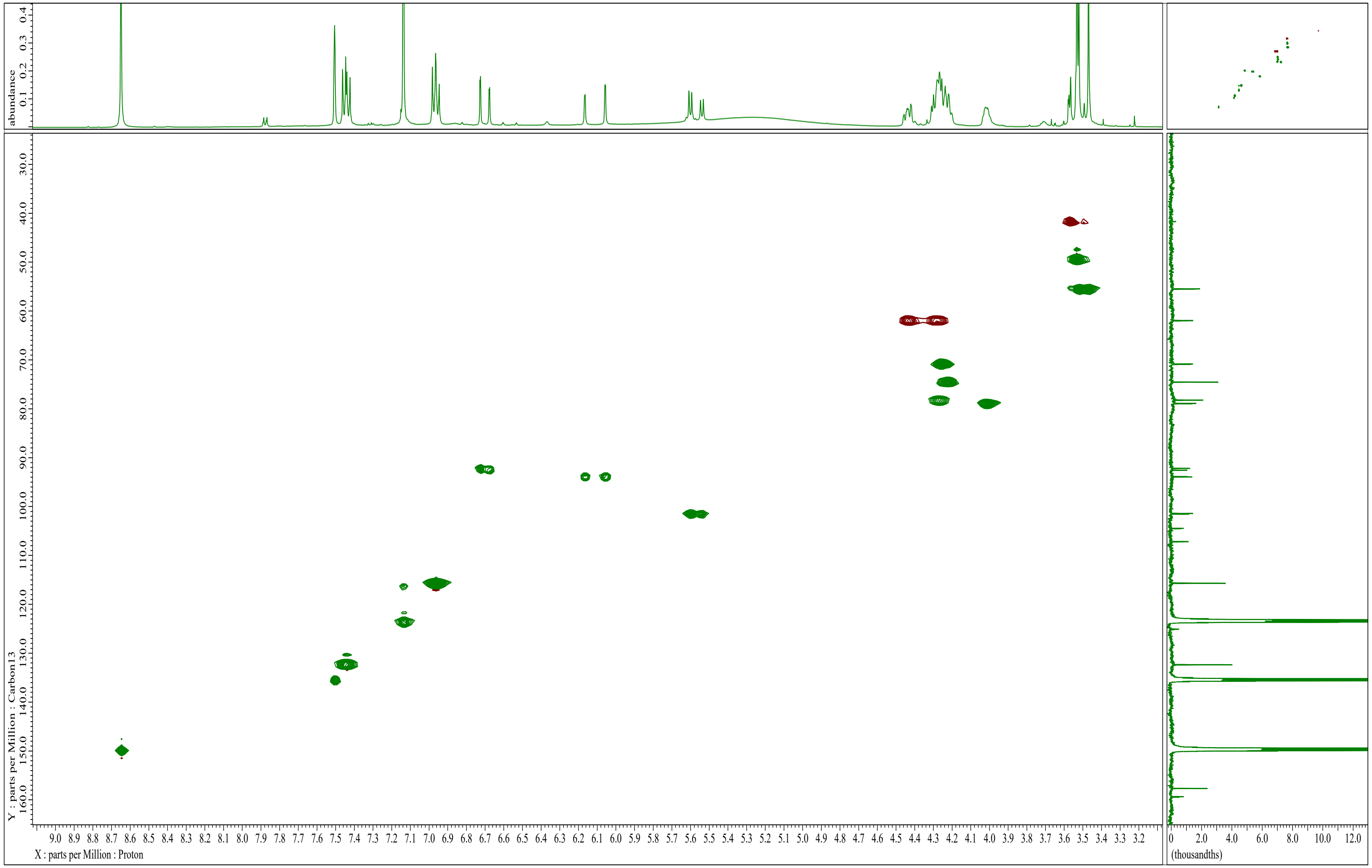

Figure S19. The COSY spectrum of compound **5** in pyridine-*d*5

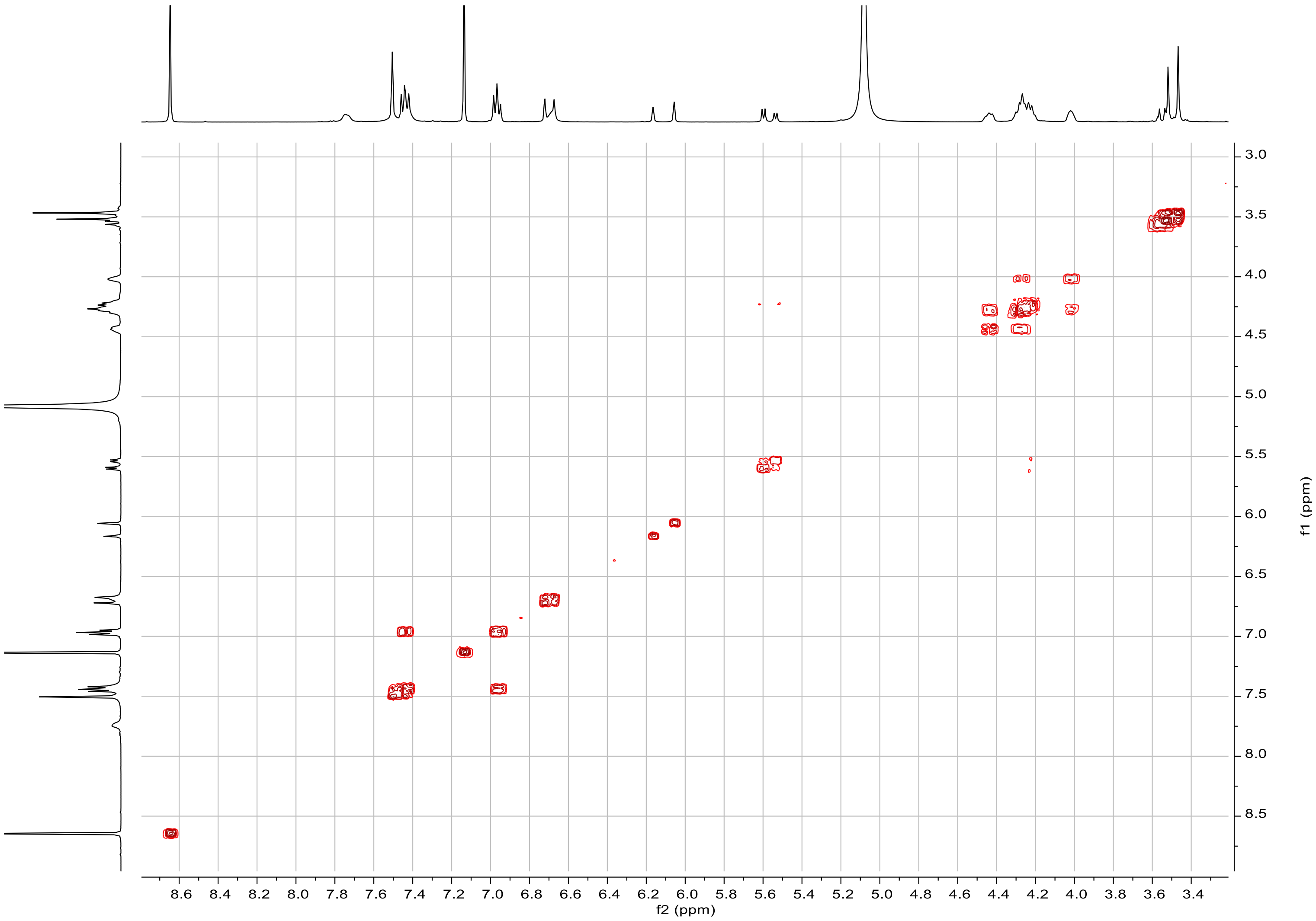

Figure S20. The HMBC spectrum of compound **5** in pyridine-*d*5

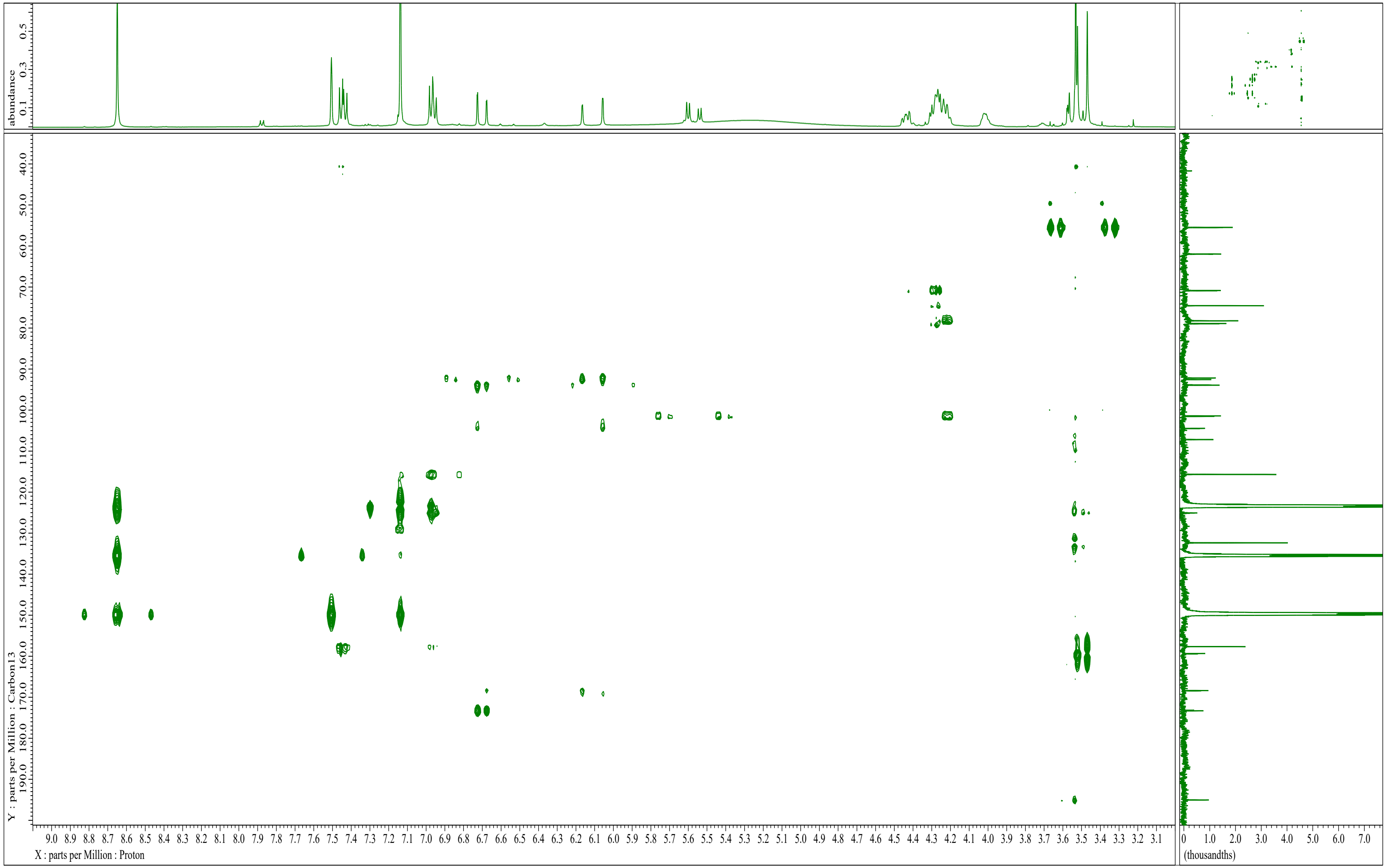

Figure S21. The NOESY spectrum of compound **5** in pyridine-d_5

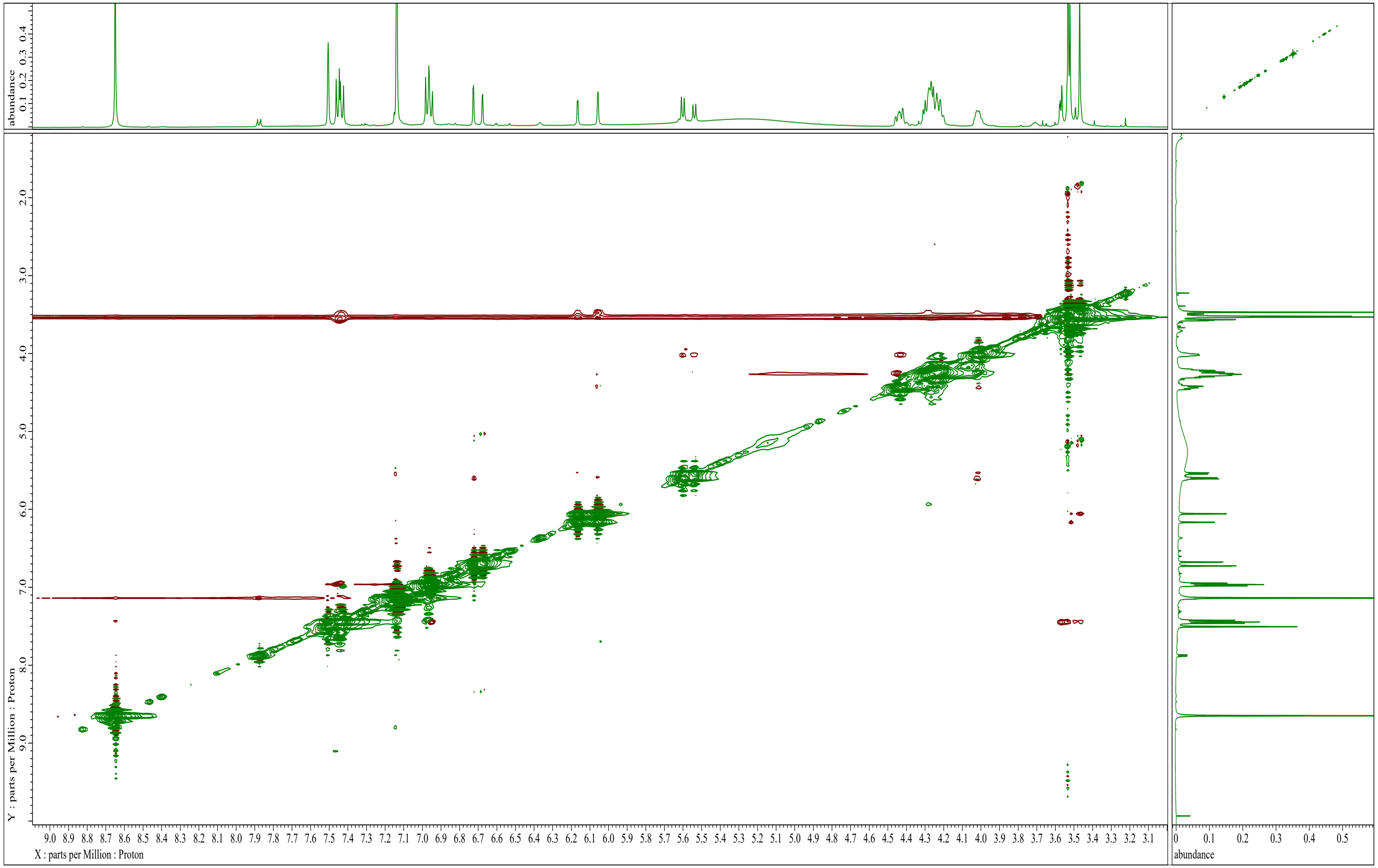

Figure S22. The IR spectrum of compound **1**

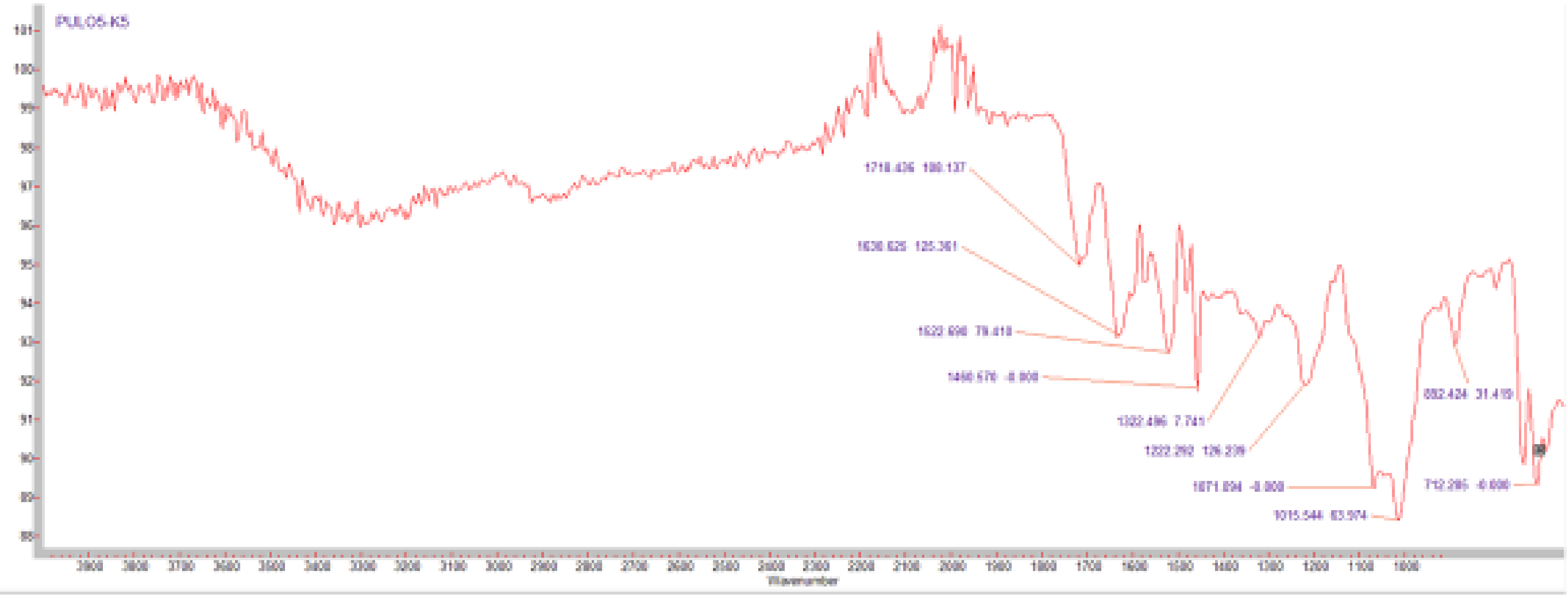

Figure S23. The IR spectrum of compound **4**

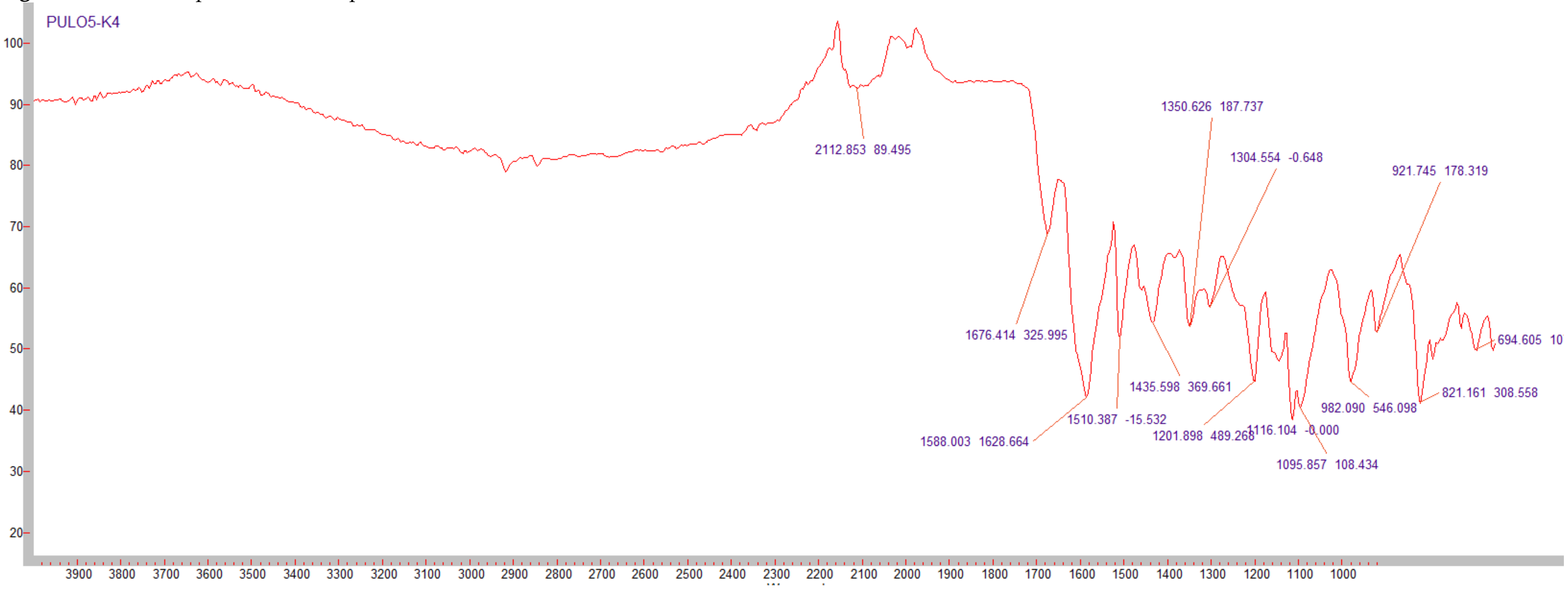

Figure S24. The IR spectrum of compound **5**

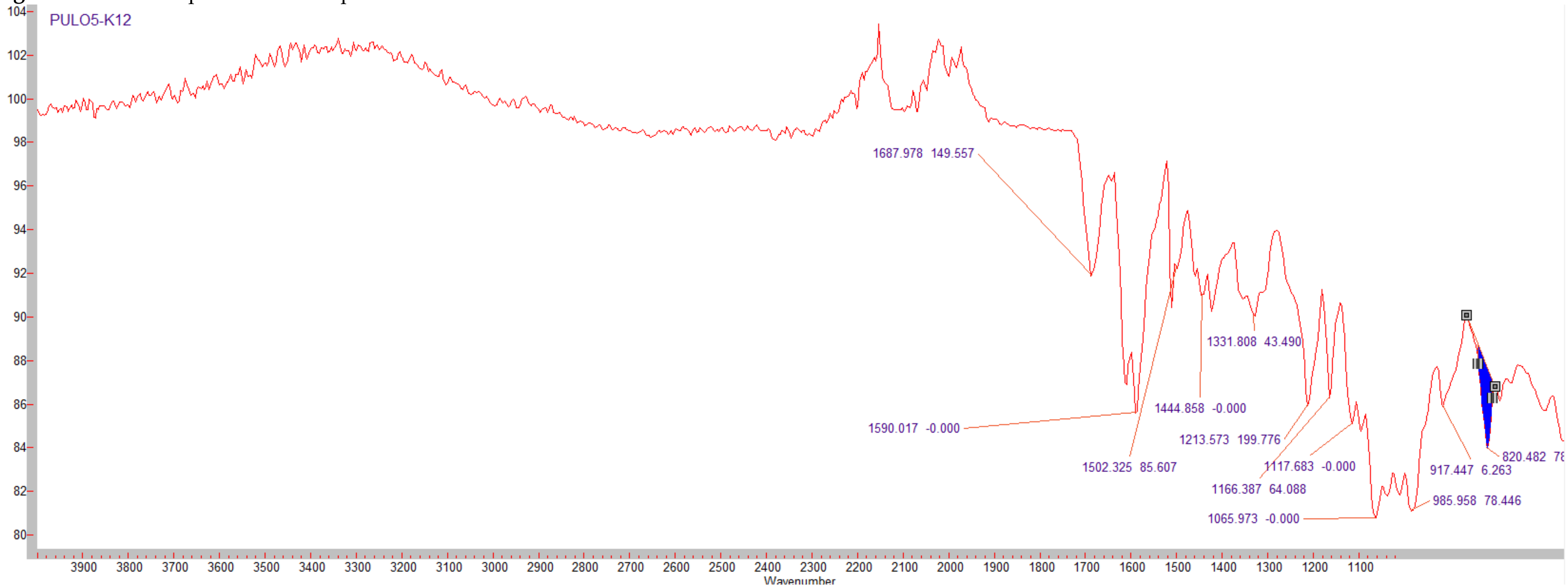

Figure S25. Analysis of sugar by HPLC (A) hydrolyzed sugar part of compound **1**; (B) D-glucose; (C) L-glucose.

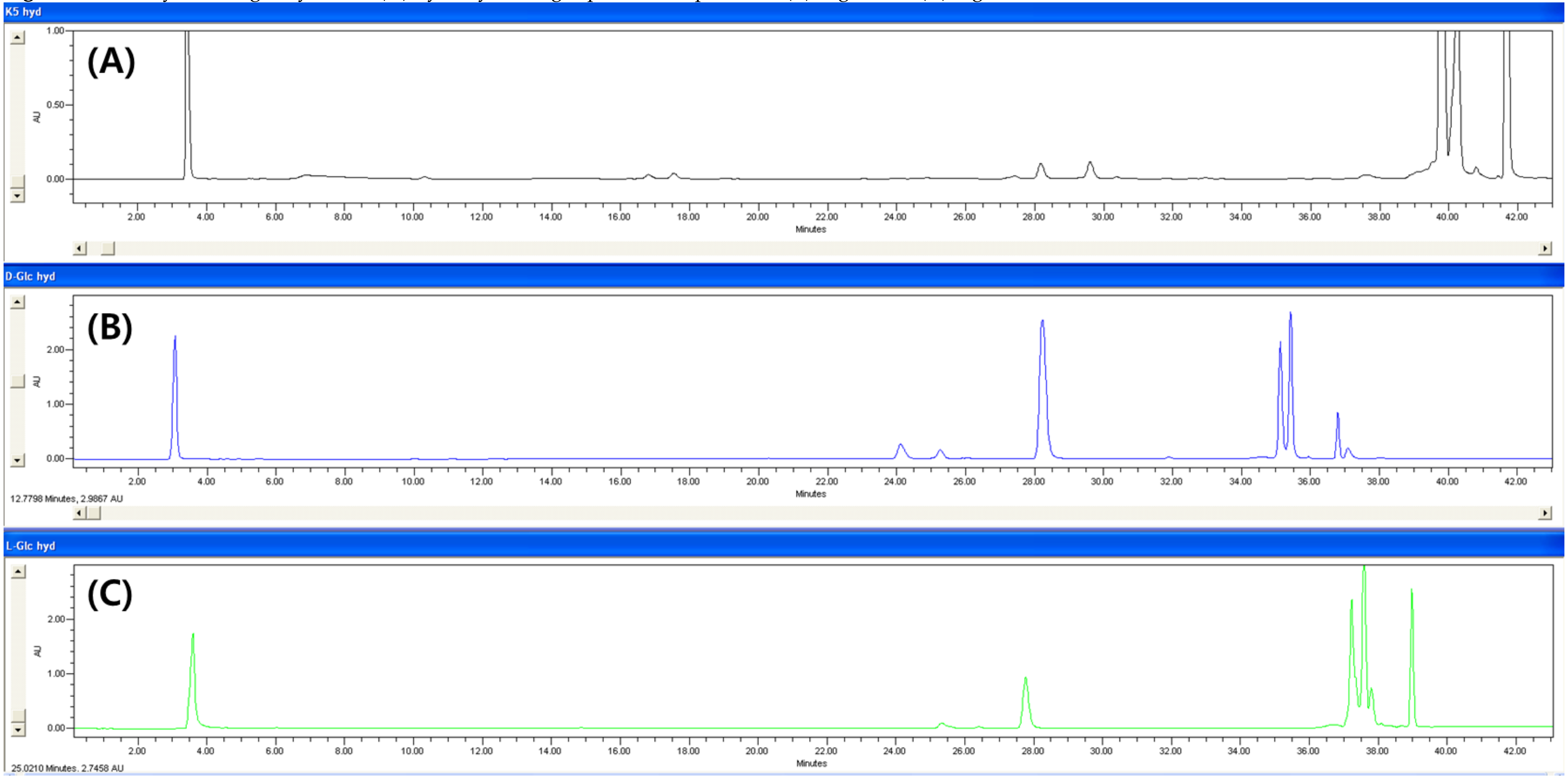

Figure S26. Analysis of sugar by HPLC (A) hydrolyzed sugar part of compound **5**; (B) D-glucose; (C) L-glucose.

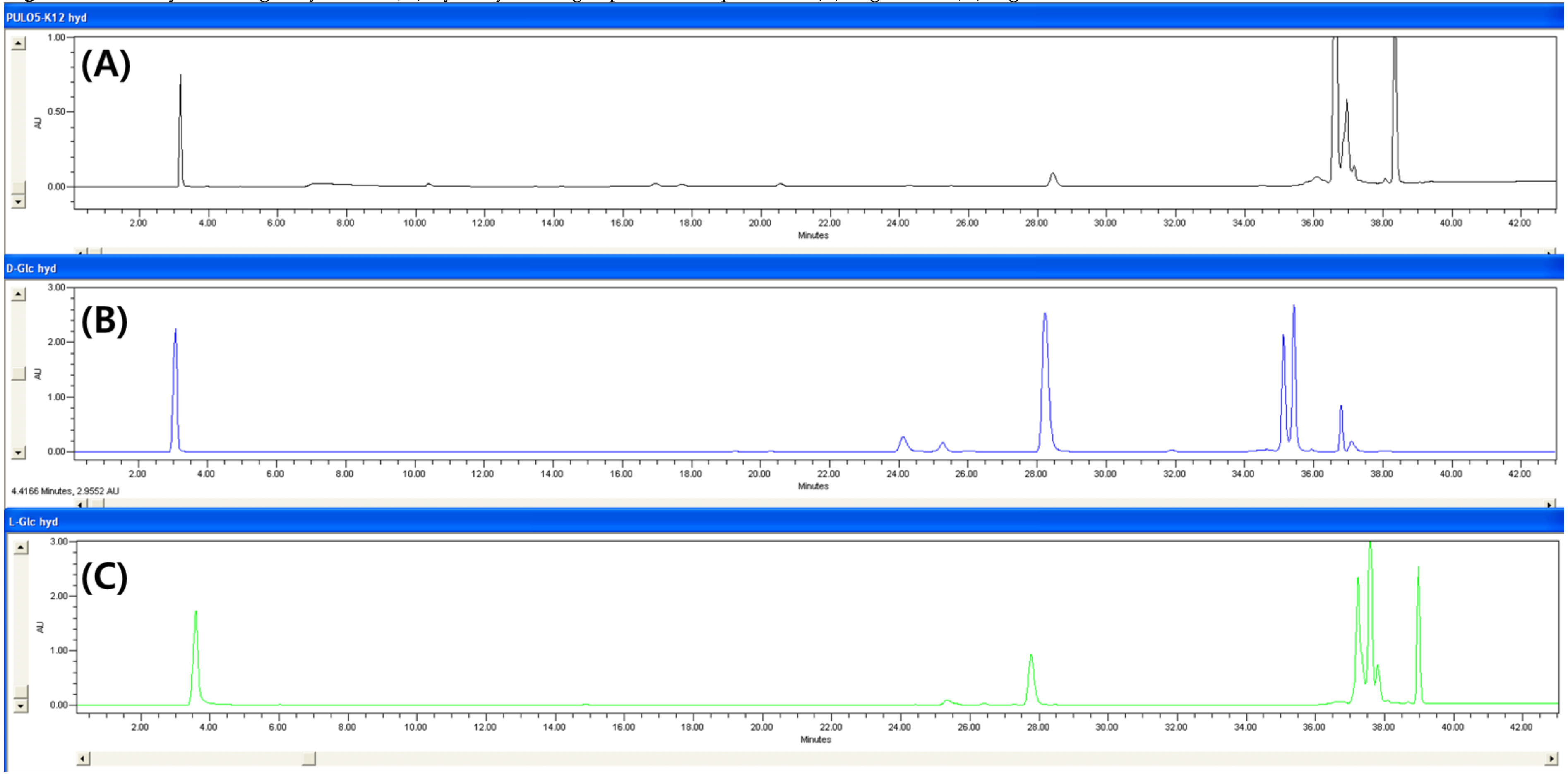

Supplement: Supplementary file 1 [file plants-11-01651-s001.zip › plants-1771004-supplementary-R2.pdf]
